# Fine-Grained Human Activity Recognition in Domestic Settings Using Ultra-Wideband Radar: A Comparative Study of Deep Learning Models

**DOI:** 10.3390/s26216714

**Published:** 2026-10-23

**Authors:** Muhammad Amjad Raza, Nasir Mehmood, Hafeez Ur Rehman Siddiqui, Adil Ali Saleem, Lázaro Javier Hernández Rodríguez, Pablo Herrero García, Isabel de la Torre Díez

**Affiliations:** 1 Institute of Computer Science, Khwaja Fareed University of Engineering and Information Technology, Abu Dhabi Road, Rahim Yar Khan 64200, Pakistan; ch.amjadraza@gmail.com (M.A.R.); nasir.mehmood@kfueit.edu.pk (N.M.); adilalisaleem@gmail.com (A.A.S.); 2 Departamento de Soluciones Tecnológicas y Sistemas, Escuela Politécnica Superior, Universidad Europea del Atlántico, Isabel Torres 21, 39011 Santander, Spain; lazaro.hernandez@uneatlantico.es (L.J.H.R.); pablo.herrero@uneatlantico.es (P.H.G.); 3 Departamento de Ciencias de la Computación, Universidad Internacional Iberoamericana, Campeche 24560, Mexico; 4 Department of Computer Science, Universidad Internacional Iberoamericana, Arecibo, PR 00612, USA; 5 Departamento de Ciencias de la Computación, Fundación Universitaria Internacional de Colombia, Bogotá 110911, Colombia; 6 Departamento de Ciências da Computação, Universidade Internacional do Cuanza, Cuito, Angola; 7 Departamento de Ciencias de la Computación, Universidad de La Romana, La Romana 22000, Dominican Republic; 8 Department of Signal Theory, Communications and Telematics Engineering, University of Valladolid, 47011 Valladolid, Spain

**Keywords:** human activity recognition, ultra-wideband radar, deep learning, transformer, convolutional neural network, fine-grained activities, ensemble learning, smart healthcare, sedentary activities

## Abstract

Human activity recognition with ultra-wideband (UWB) radar offers contactless, privacy-preserving monitoring of daily living, but distinguishing fine-grained activities performed in the same posture remains difficult. We recorded nine daily activities from 30 participants with a single PulsON 410 radar (3.1–5.3 GHz); seven are performed seated in the same chair and differ only by subtle hand and arm motions. Each 15 s recording is a 120×1440 channel–impulse–response matrix, preprocessed by background subtraction, two-dimensional wavelet denoising and standardization. Six deep learning models (Transformer, CNN-LSTM, 1D-CNN, LSTM, GRU and MLP) and a soft-voting ensemble were compared with participant-independent splits (22 training, 3 validation and 5 test participants) and five-fold cross-validation. The Transformer reached the highest cross-validation accuracy (97.74±0.30%). On the held-out participants, the Transformer (97.16%), 1D-CNN (96.93%) and GRU (96.45%) performed similarly, ahead of the CNN-LSTM, LSTM and MLP (87.47%), and a three-model ensemble reached 98.35%. Two additional participant splits and a retrained re-run confirmed that the MLP is clearly the weakest model, but the order of the other models changed by several points between runs; in the re-run, an ensemble of all six models did not exceed the Transformer (97.74%), and a range–Doppler baseline reached only 50.63%. Eating versus drinking tea and mobile phone use versus reading or sitting idle were the most confused pairs. Given the small test cohort and the controlled setting, the results are indicative rather than definitive.

## 1. Introduction

Human activity recognition has become a cornerstone technology in numerous application domains, including healthcare monitoring [1], ambient assisted living [2], smart home automation [3], and exercise recognition [4]. An automatic detection and classification of human activities can help intelligent systems to automatically intervene, adapt, and monitor the health of users during routine activities; IR-UWB radar has also been used for contactless physiological monitoring during physical activity [5]. Previous methods have focused on wearable sensor solutions, typically accelerometers and gyroscopes [6] or vision-based solutions with cameras and depth sensors [7]. However, user participation is mandatory for wearable devices and may also be uncomfortable for long-term wear, and camera-based systems may be affected by lighting and occlusion; radar sensing has been investigated for through-wall human activity classification [8].

An attractive alternative for contactless recognition of activities is offered by the use of radio-frequency sensing, and especially ultra-wideband (UWB) radar [9,10]. UWB radars are designed with very short pulse duration over a broad frequency band (usually 3.1–10.6 GHz), which allows a very high temporal resolution and very accurate range estimation [11]. Unlike optical sensors, ultra-wideband signals can go through non-metallic objects such as walls and furniture [12]. Other advantages of radar-based systems include the ability to preserve user privacy since it does not record any identifiable visual data, an important motivation for radar-based assisted living [9,13], while radar sensing does not require participants to wear devices; distributed radar networks have also been investigated for continuous activity recognition [14].

The combination of ultra-wideband radar and deep learning has led to considerable progress in recognition performance [13,15]. Unlike previous approaches in machine learning, deep neural networks can learn hierarchical representations of features directly from raw or minimally preprocessed sensor data [16]. This end-to-end paradigm is especially useful for radar data because its spatiotemporal structure is too intricate to be easily described by hand-designed features [17].

**Why fine-grained seated activities are difficult.** Most radar studies target activities that differ in gross body displacement (walking, falling, standing up), which produce strong, well-separated range–time signatures. In contrast, activities such as eating, reading a book, using a mobile phone or a laptop, drinking tea, sitting idle and putting on shoes are performed in the same seated posture, with the torso nearly stationary. Their radar signatures differ only through small-amplitude, localized hand and arm movements whose reflected energy is weak compared with static clutter and noise, and whose timing is irregular (e.g., page turning while reading). In our data, the cross-class Euclidean distances among these activities are the smallest in the dataset (Section 3.2), so inter-class separation in the raw signal space is minimal, while inter-person differences in movement style add further within-class variability. Such activities are nevertheless clinically meaningful for elderly monitoring, because changes in daily routines (e.g., eating, reading, device use) are early indicators of functional or cognitive decline.

**Differences from previous radar-based studies.** Compared with earlier UWB work (Section 2), this study (i) concentrates on activities that share one seated posture (seven of nine classes) rather than on postures or gross motions; (ii) uses a single monostatic radar at a fixed position rather than multiple or distributed radars; (iii) feeds the networks directly with preprocessed channel impulse response (CIR) matrices instead of hand-crafted Doppler or time–frequency images; (iv) evaluates all models under a participant-independent protocol with a held-out cohort in addition to cross-validation; and (v) reports ablations of the preprocessing pipeline.

Despite progress, several research gaps remain. First, most existing works focus on gross motor activities involving substantial body displacement, with limited attention to fine-grained sedentary activities that are particularly relevant for elderly monitoring and cognitive assessment. Second, comparisons of multiple deep learning architectures under a consistent, participant-independent protocol are scarce. Third, the potential of Transformer-based architectures for radar-based activity recognition remains comparatively underexplored. The main contributions of this work are:

- A dataset of nine daily activities from 30 participants (15 male and 15 female, aged 18–45 years) recorded with a single UWB radar and quality-filtered to 2658 samples, including seven fine-grained activities performed in the same seated position.
- A systematic comparison of six deep learning architectures (and a soft-voting ensemble of the three best models selected on validation data) on this task, evaluated with both five-fold cross-validation and a held-out set of unseen participants, with confidence intervals and statistical tests that account for the limited number of folds and test participants.
- An analysis of per-class and per-sample errors showing which fine-grained activities remain confusable and how far the errors of different models overlap, and of the training behavior of the models, including gradient-norm measurements of the short-lived validation collapses of the recurrent models.
- A preprocessing pipeline of background subtraction and two-dimensional Symlet-5 wavelet denoising, with ablations showing that background subtraction is the most important component and that wavelet denoising adds a smaller gain (+4.53 percentage points over no denoising in the Transformer cross-validation ablation), while an additional one-dimensional wavelet stage brought no further gain in a supplementary comparison.

**Organization of the paper.** Section 2 reviews related work and compares it with this study by activity type, radar configuration and input representation. Section 3 describes data collection, signal preprocessing, the deep learning models and the training and evaluation protocol. Section 4 presents cross-validation and held-out results, training dynamics, error analysis, computational cost, statistical analysis, ablation studies and a comparison with previous work. Section 5 discusses the findings, practical deployment in domestic environments and the limitations of the study, and Section 6 concludes the paper.

## 2. Literature Review

Among deep learning architectures, several have demonstrated notable success in sequence modeling tasks relevant to activity recognition. Long short-term memory networks [18] address the vanishing gradient problem [19] through input, forget, and output gates [20], enabling selective retention of information across extended temporal horizons [21]. Gated recurrent units [22] offer a simplified alternative with fewer parameters by merging the forget and input gates into a single update gate [23]. Convolutional neural networks, originally developed for image recognition [24,25,26], have been adapted to one-dimensional sensor signals by applying filters along the temporal dimension [6], and hybrid convolutional–recurrent architectures combine local feature extraction with sequential modeling [10,15]. The Transformer [27] replaces recurrence entirely with self-attention, which builds on earlier attention mechanisms [28] and allows each position in a sequence to attend directly to all other positions, enabling parallel computation and effective modeling of long-range dependencies. First established in natural language processing, including BERT [29], Transformers have since succeeded in other domains, including computer vision [30].

Recent research has applied these approaches to ultra-wideband radar activity recognition. Zhang et al. [5] developed a contactless system that estimates breathing and heart rate during physical activity with two IR-UWB radars, reaching an average heart-rate estimation accuracy of 86.9%. Wang et al. [8] proposed a graph convolutional network-enhanced multidomain fusion network for through-wall recognition, reporting through-wall human activity classification results. Bouchard et al. [3] used a random forest to recognize 15 activities with 80% accuracy, and Maitre et al. [10] reported 94% CNN–LSTM test accuracy with three radars, rising to 95% with voting across radar predictions. Du et al. [15] proposed a segmented convolutional gated recurrent network for classifying activity-related micro-Doppler spectrograms, while Qi et al. [11] achieved 98% accuracy for 12 activities by combining k-nearest neighbors with GoogLeNet. Lafontaine et al. [31] demonstrated a 21.5-percentage-point improvement using convolutional autoencoders for denoising, Wang et al. [12] proposed a complex-valued capsule network for through-wall activity classification, and Seyfioğlu et al. [32] showed that radar-based classification of highly similar activities benefits from learned rather than hand-crafted features. Beyond activities of daily living, domain-specific architectures have also been proposed for UWB radar gesture recognition, for example a range–Doppler dual-channel fusion vision transformer for traffic gestures [33]; such designs operate on range–Doppler image representations and target large, fast hand gestures.

Hu et al. [34] showed that impulse-radio UWB radar can detect subtle eye-blink motion without a camera, illustrating the sensitivity of radar sensing to fine-grained movements beyond gross posture changes. In a different sensing modality, Bie et al. [35] investigated multi-timescale motion recognition using tunable neuromorphic computing; their findings motivate attention to temporal scale, but are not directly comparable with UWB radar activity-recognition accuracy.

**Focused comparison.** Table 1 compares the cited studies by activity category, radar configuration and input representation, since reported accuracies are only meaningful relative to these factors. Three observations follow. (i) Almost all studies address gross-motion or posture-level activities (walking, standing, falling, lying) or physiological signals; none of the cited works isolates a set of activities performed in one identical seated posture. (ii) The better-performing activities-of-daily-living systems rely on several radars [10,14], which increases cost and installation effort. (iii) Many systems convert the radar data into Doppler, time–frequency or multi-domain images before classification, whereas the present study classifies preprocessed raw CIR matrices directly, so that the temporal structure is exploited by the network itself.

There are a few caveats, however. Current systems often use several radar units, which complicates deployment and raises hardware cost. The reported models are able to reach good accuracy for activities that are very different in body posture, but evidence for fine-grained sedentary tasks that require nearly the same seating posture is limited. Recurrent mechanisms can be unstable when used on long radar sequences, and preprocessing mechanisms tend to be under-explored and may not be robust in noisy indoor environments. To address these challenges, the present study investigates attention-based, convolutional, recurrent, and ensemble models and evaluates their performance on a dataset that contains very similar activities, using a wavelet-based preprocessing pipeline and a single radar sensor.

## 3. Methodology

**Figure 1:**
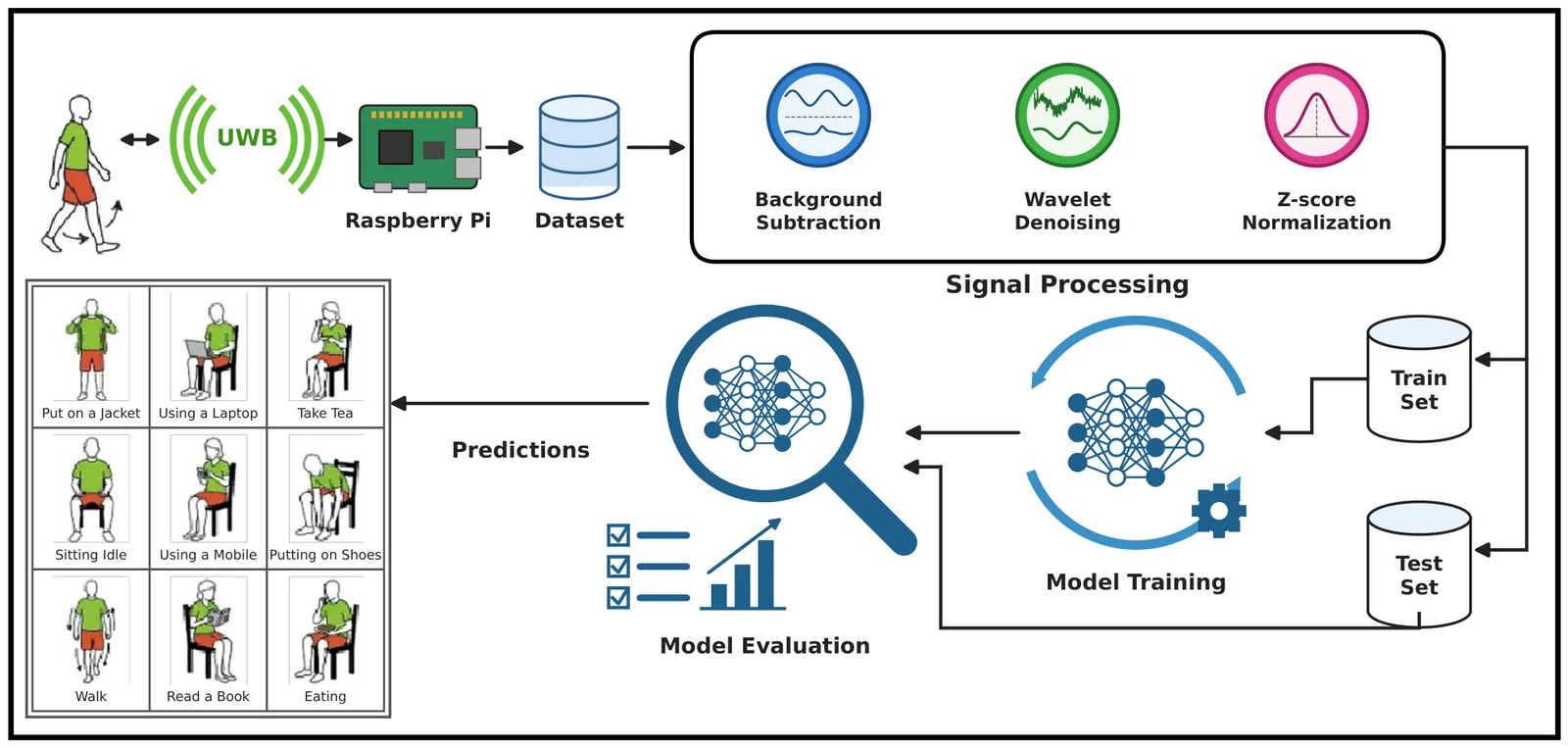
Proposed methodology for ultra-wideband radar-based human activity recognition. The Raspberry Pi is used only to acquire and store the radar data; preprocessing, model training and evaluation are performed offline (Section 3.4).

The recognition framework follows a structured pipeline that integrates data acquisition, signal preprocessing, and deep learning-based classification, as shown in Figure 1. Raw radar measurements capture the time-varying reflections generated by human movement; they are processed to suppress background clutter and noise and are then standardized before being passed to the classification models. This modular design enables systematic assessment of the contribution of each component to recognition performance.

### 3.1. Data Collection

**Figure 2:**
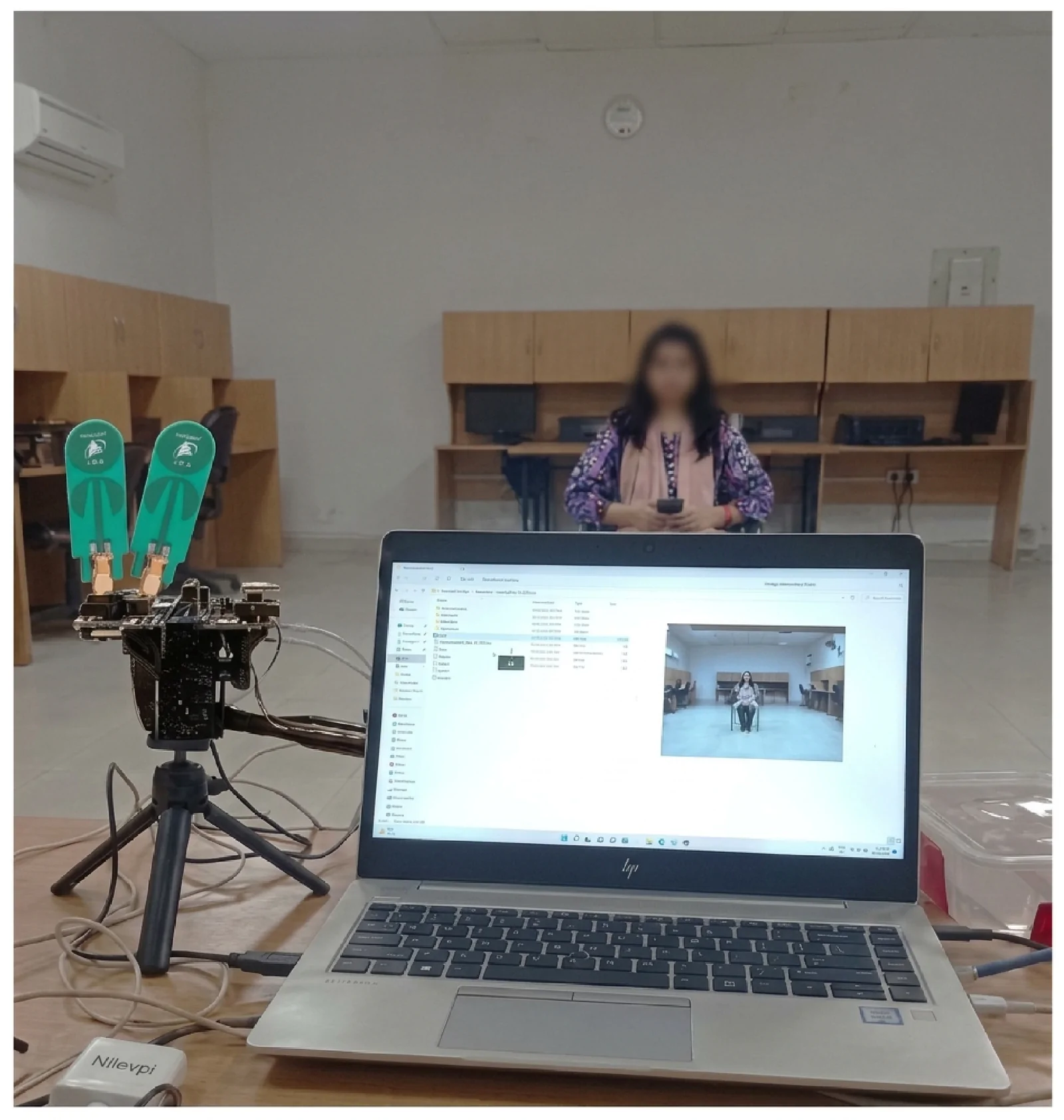
Data collection setup.

The dataset was collected using a Time Domain PulsON 410 (P410) monostatic ultra-wideband radar module [36] equipped with a single omnidirectional transmit antenna and a single omnidirectional receive antenna and compliant with Federal Communications Commission (FCC) regulations for ultra-wideband devices. The radar transmits within the 3.1–5.3 GHz band, with a center frequency of 4.3 GHz and a bandwidth of 2.2 GHz, corresponding to a fractional bandwidth of 51.16%, which exceeds the FCC requirement for ultra-wideband operation. The module operated with a transmit gain of −12.64 dBm, an effective range of 9.5 m and a recording duration of 15 s per sample, and is rated for operation between 0 and 70 °C. The radar was mounted at a height of 72 cm and oriented toward the activity region, and participants performed all activities at a distance of 2 m from the radar as shown in Figure 2. The radar was connected to a Raspberry Pi, which served only as the acquisition unit: it controlled the radar and stored the recorded scans. All preprocessing, training and evaluation were carried out offline on the recorded data in a cloud environment (Section 3.4); no model was trained or run on the Raspberry Pi.

**Figure 3:**
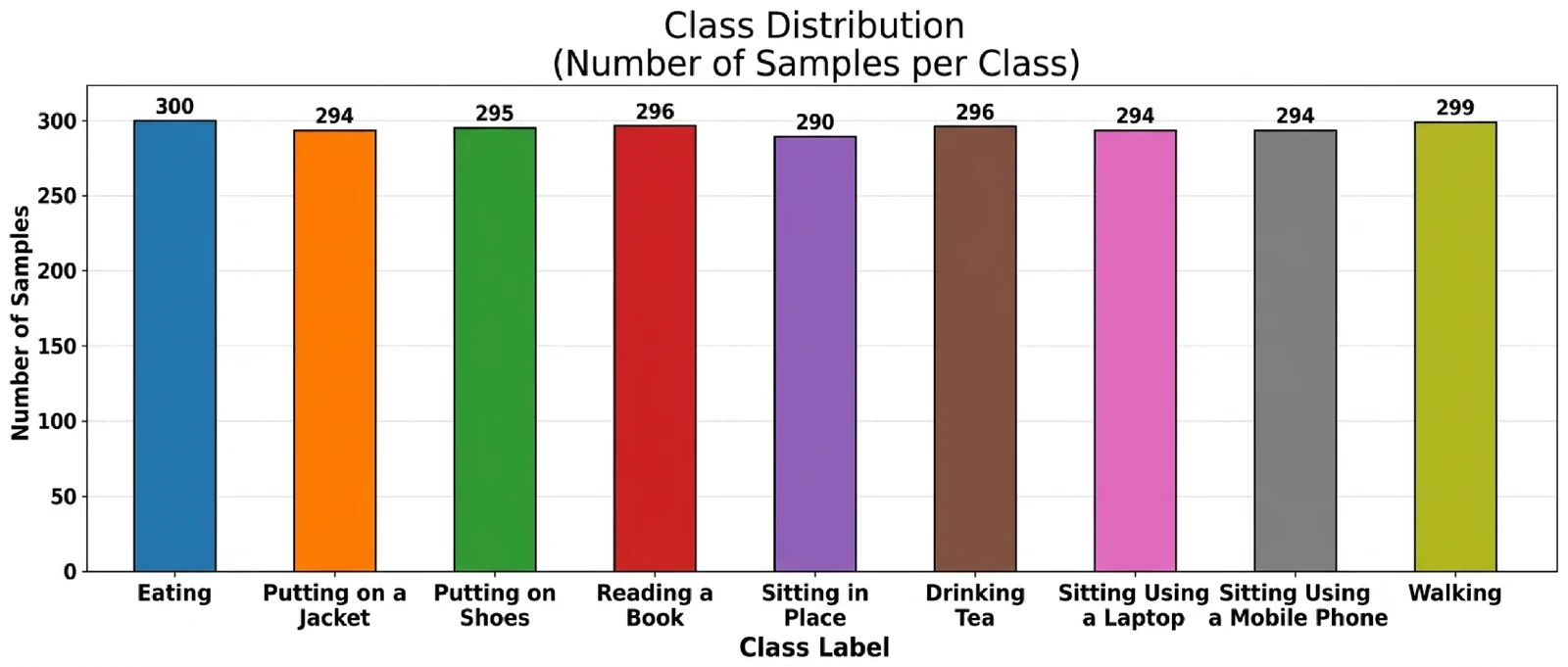
Class distribution across the nine activities after quality filtering.

The thirty healthy adult volunteers (aged 18–45 years, 15 men and 15 women) agreed to participate after having been informed accordingly in compliance with institutional ethical standards. Nine activities, from fine-grained hand movements to full-body movement, were conducted by each participant. Walking and putting on a jacket required larger body displacement than the other seven activities (eating, putting on shoes, reading a book, sitting in place, drinking tea, sitting using a laptop and sitting using a mobile phone), which were executed while sitting on the same chair at a fixed position. All activities were videotaped (for labeling and quality control) and repeated over several sessions of 15 s each. All recordings were checked; recordings from sensors with malfunctions, corrupted readings, or irregular signal patterns were eliminated. The dataset has 2658 samples, resulting from a quality filtering process which left an approximately even distribution over the nine activities (Figure 3).

**Input matrix and acquisition settings.** Each sample is one 15 s recording stored as a matrix $X\in R^{T\times R}$ with T=120 rows and R=1440 columns. The rows are successive radar frames along *slow time*: the 120 frames span the 15 s recording, i.e., a frame interval of 15/120=0.125 s (8 frames per second) (the radar’s native scan rate is not used directly; each stored sample contains exactly 120 frames). The columns are *fast-time* samples of the channel impulse response, i.e., range bins; the 1440 bins cover a range window of 9.3 m, which gives a bin spacing of 9.3/1440≈0.65 cm and includes the activity region at 2 m from the radar. The 9.3 m window lies within the 9.5 m effective range of the module. Recordings whose stored matrix did not have exactly the shape 120×1440 were excluded when the data were loaded. Each row therefore describes the reflections from all ranges at one instant, and each column follows the reflectivity of one range bin over time.

**Figure 4:**
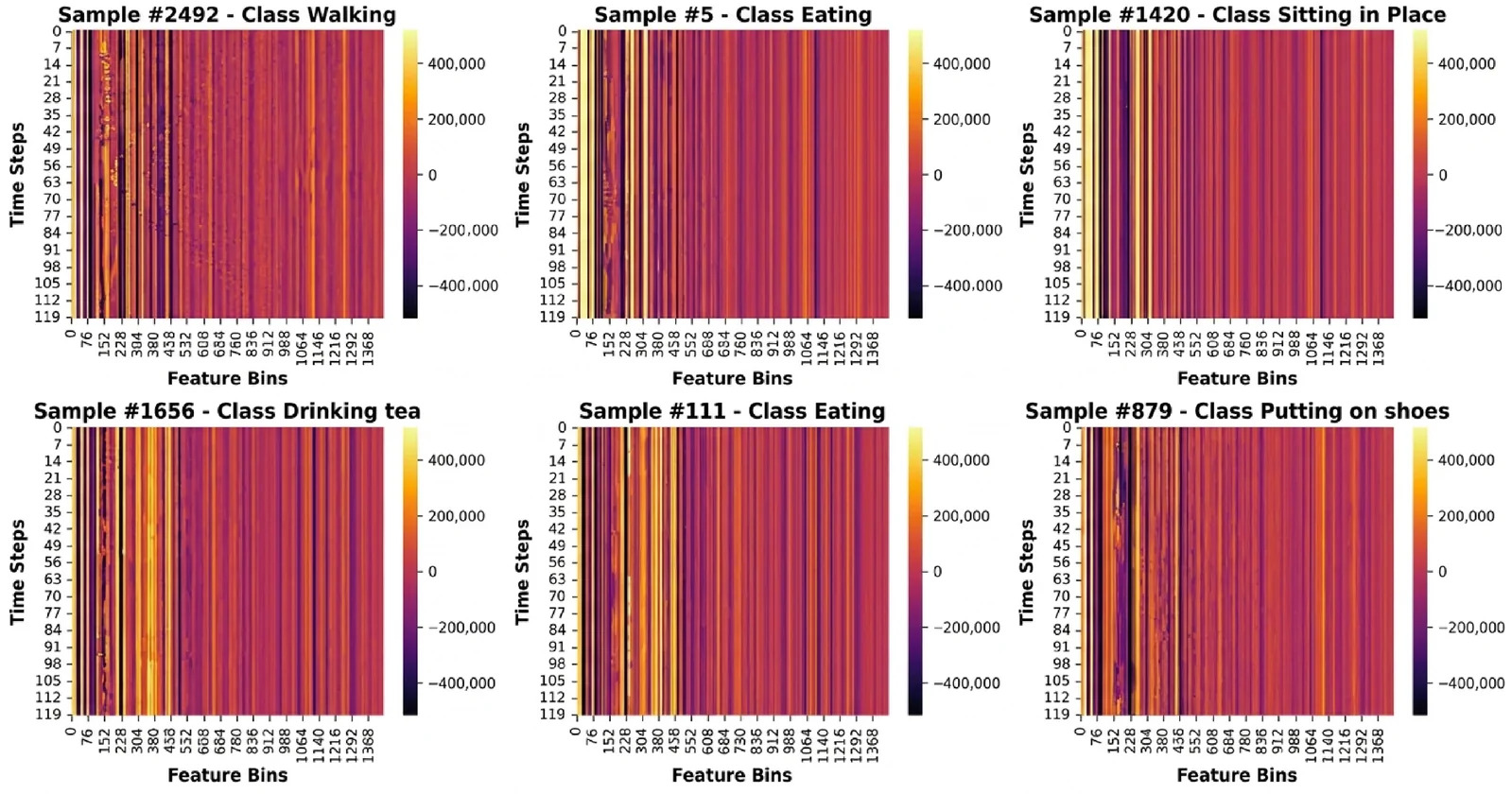
Representative radar signal matrices (120×1440) for different activity classes.

Figure 4 shows some typical radar matrices. While walking leaves behind distinct diagonal patterns, tasks while sitting, e.g., laptop use or putting on shoes, have subtle variations focused within specific range regions. The fine-grained classification challenge tackled in this work is well captured in the strong visual similarity between the activities that involve sitting.

### 3.2. Signal Preprocessing

All radar samples pass through a preprocessing pipeline consisting of background subtraction, wavelet denoising, and normalization. To suppress static clutter from walls, furniture, and other fixed objects while preserving motion-induced variations, an exponential-moving-average background estimate B(t) is maintained along slow time (frame index *t*, independently for each range bin) and subtracted from each frame:

(1) $$B\left(t\right)=\alpha B\left(t-1\right)+\left(1-\alpha \right)X_{\mathrm{raw}}\left(t\right),\;X_{\mathrm{clean}}\left(t\right)=X_{\mathrm{raw}}\left(t\right)-B\left(t\right),$$

where α=0.95 is the smoothing factor controlling temporal persistence.

Wavelet denoising is then applied to suppress high-frequency noise originating from hardware imperfections and multipath disturbances [37]. A Symlet-5 (sym5) wavelet [38] is used; symlets are the near-symmetric members of the Daubechies family, and their near-linear phase limits the distortion of short transient echoes. The two-dimensional discrete wavelet transform (PyWavelets wavedec2) is applied to the whole 120×1440 matrix (slow time × fast time) with the sym5 wavelet and a decomposition level of 2. All detail sub-bands are soft-thresholded using a universal threshold [39] that is scaled by a multiplier of 1.5 (moderate denoising),

(2) $$\tau =1.5\;{\hat{\sigma }}_{n}\sqrt{2\ln N},$$

where ${\hat{\sigma }}_{n}$ is the noise standard deviation estimated from the median absolute deviation of the finest-scale (level-1) horizontal detail coefficients divided by 0.6745, and N=120×1440=172,800 is the number of samples of the matrix. The approximation coefficients are left unchanged. The transform is applied to the background-subtracted matrix and uses the symmetric boundary extension of PyWavelets (its default). The denoised matrix is reconstructed via the inverse wavelet transform. Finally, the data are standardized feature-wise:

(3) $$X_{\mathrm{norm}}\left(t,r\right)=\frac{X_{\mathrm{den}}\left(t,r\right)-\mu \left(t,r\right)}{\sigma (t,r)},$$

where $X_{\mathrm{den}}$ is the denoised sample and μ(t,r) and σ(t,r) are the mean and standard deviation of element (t,r) of the 120×1440 matrix over the training samples (scikit-learn StandardScaler). The statistics are computed on the training set only and then applied unchanged to the validation and test samples, so that no information from these sets enters the preprocessing. The wavelet family, decomposition level and thresholding multiplier were chosen empirically; the effect of the wavelet stage was re-examined in a supplementary experiment (Section 4.8).

**Figure 5:**
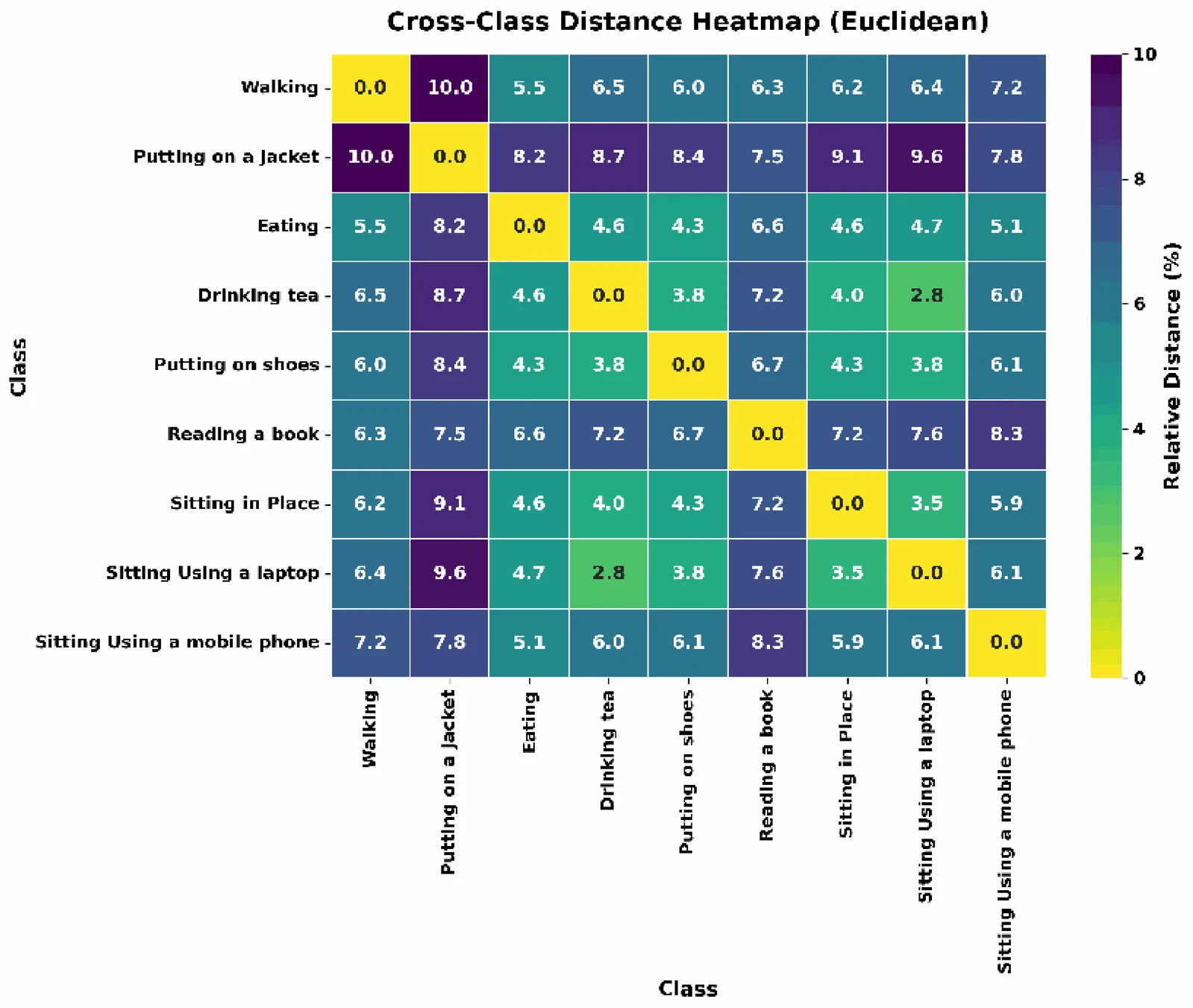
Cross-class Euclidean distance matrix showing low separation between most activity pairs.

The difficulty of the classification problem is illustrated in Figure 5, which shows the cross-class Euclidean distance matrix computed from the raw radar signals. Distances are low (2.8–4.7) among activities performed in similar seated positions, such as drinking tea, putting on shoes, sitting idle, and using a laptop. Reading a book and using a mobile phone are somewhat more distant from sitting idle (7.2 and 8.3), and walking records distances of only 5.5–7.2 to most seated activities, confirming the overall lack of separation in the raw signal space. Only putting on a jacket clearly stands apart (7.5–10.0), owing to its larger full-body movements. This limited inter-class separation motivates models capable of learning subtle temporal dependencies.

### 3.3. Deep Learning Architectures

Six deep learning architectures and one ensemble model were evaluated. Each model receives a preprocessed 120×1440 radar matrix, treated as a sequence of 120 time steps with 1440 features, and outputs a probability distribution over the nine activity classes. Table 2 summarizes their configurations. The six architectures were chosen to represent the main model families used for radar time series (feed-forward, convolutional, recurrent, hybrid convolutional–recurrent, and attention-based), so that the study isolates which inductive bias suits raw CIR sequences; they are generic baselines and not specialized UWB-gesture networks (see Section Comparison with Domain-Specific UWB Gesture/Activity Architectures).

The Transformer uses an encoder-only design: a linear layer projects each of the 120 time steps (1440 features) to a $d_{\mathrm{model}}$-dimensional space, learnable positional encodings are added, and a stack of standard Transformer encoder layers (multi-head self-attention, layer normalization, ReLU feed-forward network, residual connections, dropout 0.1) is followed by global average pooling over time and a linear classifier. The proposed configuration uses $d_{\mathrm{model}}=256$, eight attention heads, four encoder layers and a feed-forward width of 2048 (Section 4.8); the positional encodings are initialized to zero. The CNN-LSTM applies one one-dimensional convolution (128 filters, kernel size 3) over time with the 1440 range bins as input channels, followed by batch normalization and max pooling (which halves the sequence to 60 steps), and a two-layer bidirectional LSTM (256 hidden units per direction, dropout 0.2) whose final hidden states of both directions feed a linear classifier. The 1D-CNN consists of three convolutional blocks (kernel size 3, 128, 256 and 256 filters; ReLU, batch normalization, max pooling) over the time axis with the 1440 range bins as channels, followed by global average pooling and a classifier with a 128-unit hidden layer and dropout 0.5. The LSTM and GRU networks are two-layer bidirectional recurrent networks with 256 hidden units per direction and dropout 0.2 between layers; the final hidden states of both directions are concatenated and passed to a linear classifier. The MLP baseline flattens the input to 172,800 features and processes it through fully connected layers with 1024 and 512 units (ReLU, dropout 0.5) and a linear output layer; it serves as a non-sequential reference. Batch normalization [40] and dropout [41] are used for regularization as described above.

**Ensemble design rationale.** The soft-voting ensemble combines the three single models with the highest validation accuracy: the Transformer, the GRU and the 1D-CNN in the main 22/3/5 split (in the 20/3/7 and 17/3/10 splits: the CNN-LSTM, Transformer and 1D-CNN). The members were selected based on the accuracy on the validation participants only; in the main split these are the Transformer (97.53%), the 1D-CNN and the GRU (both 96.71%), ahead of the CNN-LSTM (95.88%; Section 4.2), and the test participants played no role in the selection. The members rely on different inductive biases: self-attention aggregates information across all 120 time steps (global temporal context), the gated recurrent network processes the sequence step by step with a learned memory, and the convolutional network extracts local motion patterns with shared filters before pooling. Ensembling helps only if its members make different errors, and the different biases are expected to promote this. The ensemble averages the softmax class probabilities produced by the independently trained members with equal weights and assigns the class with the highest averaged probability; it introduces no additional trainable parameters.

### 3.4. Training Configuration and Evaluation Protocol

All models were trained using the Adam optimizer [42] with a constant learning rate of $10^{-4}$ (no weight decay, learning-rate schedule or gradient clipping), batch size of 32, and categorical cross-entropy loss. Every model was trained for the full 100 epochs without early stopping; the weights of the epoch with the lowest validation loss were kept and used for testing. To prevent participant-level data leakage, the 30 participants were divided into three mutually exclusive groups: 22 participants for training, 3 for validation, and 5 for testing (approximately 75%, 10%, and 15% of the participants), so that no participant appears in more than one subset. Five-fold cross-validation was performed exclusively within the training participants for hyperparameter selection (the folds were split by participant, so that no participant appears in both the training and the validation part of a fold); the cross-validation results reported in Section 4.1 were obtained with the final configuration of each model. The test participants were never used during training or model selection, and the held-out test results in Section 4.2 therefore measure generalization to unseen individuals. Performance is reported in terms of accuracy and macro-averaged precision, recall, and F1-score on the training, validation and test participants. Because the held-out set contains only five participants (423 samples), test accuracies are accompanied by 95% Wilson score confidence intervals for a binomial proportion [43]. Differences between models are assessed with paired *t*-tests over the five cross-validation folds and with Cohen’s *d* effect sizes; since the folds share training participants and are therefore not independent, we additionally report the variance-corrected resampled *t*-test of Nadeau and Bengio [44], confidence intervals of the paired differences, and the exact Wilcoxon signed-rank/sign-test result (Section 4.7). All experiments were conducted in a Kaggle Notebook (NVIDIA Tesla T4 GPU accelerator, an Intel Xeon 2.20 GHz CPU, 30 GB of RAM) with PyTorch 2.0.

**Supplementary analyses.** Additional analyses were carried out in the same Kaggle environment (Intel Xeon 2.00 GHz CPU with four threads, NVIDIA Tesla T4 GPU, PyTorch 2.11): a sample-level analysis of the errors of the six models, training of the LSTM and GRU with gradient-norm logging and gradient clipping, a comparison of wavelet settings, a range–Doppler baseline, and CPU and GPU timing. For these analyses all six models were retrained with the architectures and hyperparameters described above (random seed 42). The re-run used a random, class-stratified split of the individual recordings into 1989 training, 266 validation and 399 test samples (75%, 10% and 15%; 44–45 test samples per class), with feature-wise standardization fitted on the training samples. Because this split is made per recording and not per participant, recordings of the same participant can occur in both the training and the test set; the re-run therefore measures recognition of new recordings rather than of unseen participants and is likely to be optimistic. Its results are reported separately (Section 4.2) and are not pooled with those of the participant-independent main split; each supplementary experiment was run once.

## 4. Results and Discussion

This section presents the cross-validation and held-out test performance of all models, the training dynamics, per-class and confusion analyses, computational cost, statistical testing, ablation studies, and a comparison with previous work.

### 4.1. Cross-Validation Performance

Table 3 reports the five-fold cross-validation accuracy of each architecture, together with the mean, standard deviation, and 95% confidence interval (Student’s *t*-distribution, four degrees of freedom). Because the classes are approximately balanced, the mean macro F1-score was within 0.01 percentage points of the mean accuracy for every model.

The Transformer achieves the highest accuracy in every fold, followed by the CNN-LSTM, 1D-CNN, LSTM, GRU, and MLP. All architectures with explicit temporal modeling outperform the MLP baseline, which is consistent with the importance of sequential processing for radar-based activity recognition, and the Transformer improves on the next-best CNN-LSTM by 1.13 percentage points.

### 4.2. Held-Out Test Set Performance and Ensemble Evaluation

Table 4 reports the performance of all architectures and of the soft-voting ensemble on the training, validation and held-out test participants (423 test samples). On the test participants, the ensemble of the Transformer, GRU and 1D-CNN achieved the highest accuracy (98.35%), followed by the Transformer (97.16%), the 1D-CNN (96.93%), the GRU (96.45%), the CNN-LSTM (95.04%), the LSTM (92.91%) and the MLP (87.47%). The Transformer and the 1D-CNN differ by a single test sample (411 versus 410 of 423 correct) and the GRU by three, so these three models form one performance tier, indicating that global self-attention, local convolutional feature extraction and gated recurrence are all effective for this task. The MLP is clearly the weakest model, which is consistent with the importance of temporal modeling. All models except the MLP (97.13%) and the LSTM (99.54%) reach 100% training accuracy, so the gap between training and validation or test accuracy reflects overfitting to the training participants, which is largest for the MLP (97.13% versus 89.30% on validation). The validation and test accuracies of each model are close (e.g., 97.53% versus 97.16% for the Transformer), which indicates that the three validation participants were representative of the test participants.

**How much can five test participants tell us?** Five participants (423 samples) are a small cohort. One misclassified sample changes accuracy by 0.24 percentage points and the binomial standard error near 97% accuracy is about 0.83 points, so differences below roughly 2 points cannot be resolved. The intervals in Table 4 make this explicit: the ensemble, the Transformer, the 1D-CNN and the GRU have strongly overlapping intervals and should be regarded as one performance tier; the CNN-LSTM overlaps only the lower end of this tier, and the LSTM and the MLP are clearly lower (their upper bounds, 94.99% and 90.29%, lie below the lower bound of the ensemble, 96.62%). The test results are therefore interpreted as a coarse grouping of the models and not as a precise ranking, and the generalization conclusions apply to the studied cohort (healthy adults aged 18–45 years, one room, one chair). Because the main test set is a single draw of participants, it does not capture the variance arising from other participant groups; two additional splits are reported below, and leave-one-subject-out evaluation on a larger cohort remains necessary future work (Section 5.2).

**Cross-validation versus test results.** Moving from cross-validation (Table 3) to the unseen test participants, the Transformer changes by −0.58 points (97.74% to 97.16%), the CNN-LSTM by −1.57, the LSTM by −1.43, the 1D-CNN by +1.08, the GRU by +3.24 and the MLP by −3.85. Changes of about one point are within the 0.83-point sampling uncertainty of the test set; only the GRU (increase) and the MLP (decrease) change by more. The Transformer leads in cross-validation and is also the best single model on the test participants, but its margin over the 1D-CNN is 1.89 points in cross-validation and a single sample on the test set, which shows that the 1D-CNN generalizes at least as well as its cross-validation score suggests. The large increase of the GRU is notable and was not investigated further. The ranking of the single models is therefore not identical in the two protocols, and we do not claim that either protocol is more reliable: cross-validation folds share training participants, whereas the test set contains unseen participants but only five of them.

**Robustness to the participant split.** To check that the conclusions do not depend on one choice of five test participants, all models were also trained and tested with two further participant-wise splits with more test participants and fewer training participants (20/3/7 and 17/3/10; Table 5). All three runs used the same hyperparameters (Section 3), and the test participants of the three splits do not overlap. The splits show the following. (i) The *ordering by model family is largely stable*: the MLP is the weakest model in every split (86.6–87.6%), and the 1D-CNN is among the three best single models in all splits, the Transformer and the GRU in two of three. (ii) The *1D-CNN is the most stable single model* (94.39–96.93%, range 2.5 points) and the best single model in two of the three splits, whereas the Transformer is the best single model on the 22/3/5 split (97.16%) but loses accuracy as the number of training participants decreases (97.16, 94.69 and 90.95%); this is consistent with attention-based models needing more training data, which we did not test. With 17 training and 10 test participants, the 1D-CNN is 3.4 points ahead of the Transformer, so the near-tie observed on the main split does not extend to every split. (iii) The Transformer (range 6.2 points) and the LSTM (5.8 points) vary most between splits. (iv) Nearly all models reach 97–100% training accuracy in every split, so absolute test accuracy depends on the participants chosen: the spread of the same model across splits is larger than the differences between many models within a single split. (v) The three-model ensemble reached the highest accuracy on the 22/3/5 and 20/3/7 splits (98.35% and 96.68%, i.e., +1.19 and +0.99 points over the best single model) and was within 0.12 points of the best single model (1D-CNN) on the 17/3/10 split (94.27% versus 94.39%). We conclude that (a) the Transformer, 1D-CNN and GRU all generalize well, but none is uniformly better, (b) the performance of any single model can change by several points between participant groups, which confirms that a five-participant test set gives only a coarse estimate, and (c) small ensembles of complementary models, selected on validation accuracy, are a promising way to reduce this variability.

**Re-run with retrained models.** Table 6 lists the test accuracies of the retrained models of the supplementary per-recording evaluation (399 test samples of a per-recording split; Section 3.4). The Transformer was again the best single model (97.74%), followed by the CNN-LSTM (96.74%), the LSTM (95.24%), the 1D-CNN (94.49%), the GRU (92.98%) and the MLP (86.47%). Compared with the main split, the Transformer and the MLP keep their positions, but the order of the other four models changes and individual accuracies differ by up to 3.5 points (GRU: 96.45% versus 92.98%). Training the LSTM and GRU once more for the clipping experiment (Section 4.3) gave 93.23% and 94.49% without clipping, i.e., the same configuration differed by 1.5–2 points between training runs on the same split. These run-to-run differences are of the same size as most differences between the convolutional, recurrent and hybrid models, which confirms that only the coarse grouping is reliable: the Transformer at or near the top, the MLP clearly lowest, and the other four models in between. Although the re-run split is not participant-independent, which would be expected to favor all models, its accuracies are of the same order as those of the participant-independent main split. The soft-voting ensemble of all six re-run models reached 96.74%, one point below the Transformer alone (Section 4.4).

### 4.3. Training Dynamics Analysis

**Figure 6:**
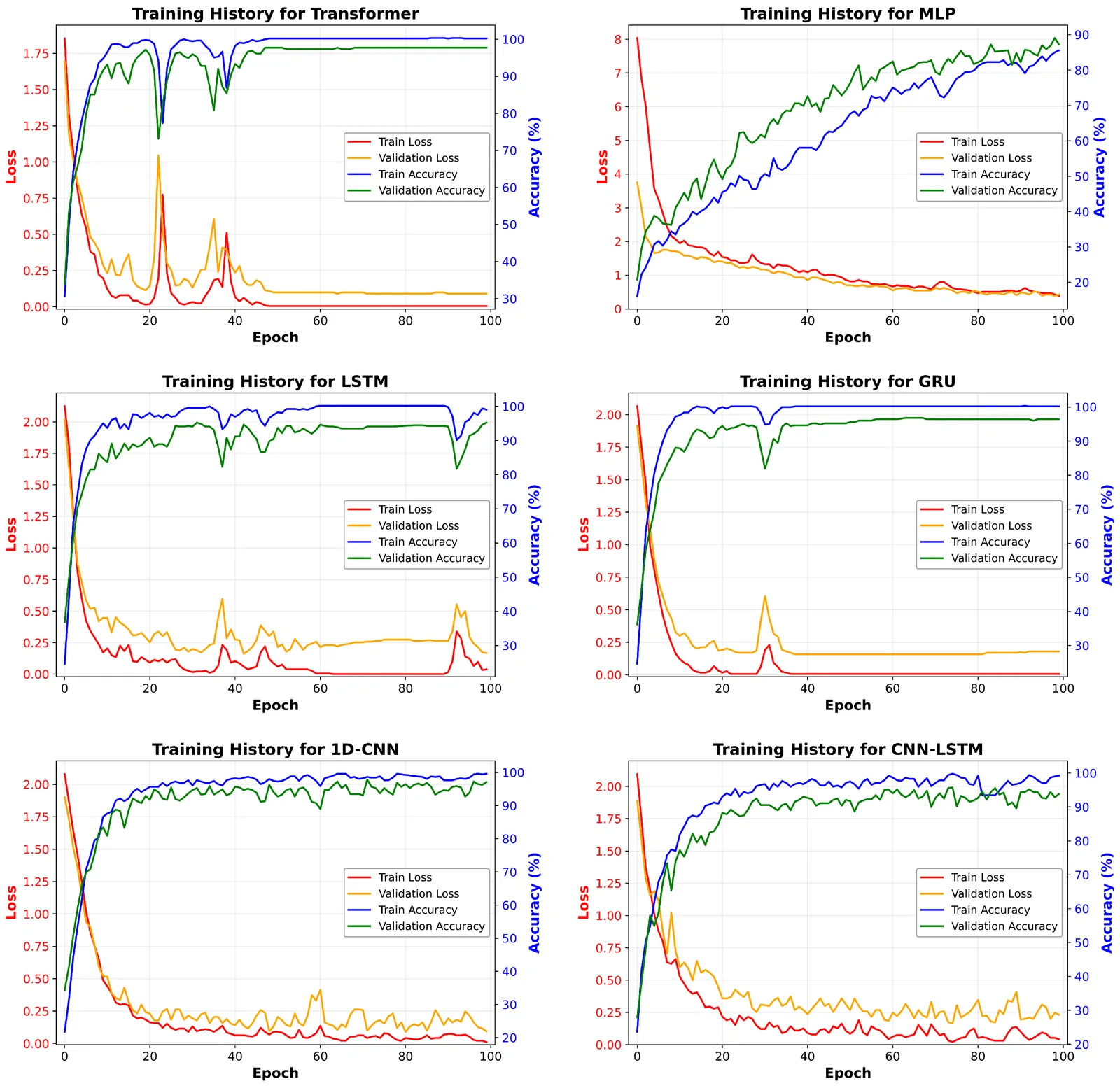
Training l history (training and validation loss and accuracy) over 100 epochs for the Transformer, MLP, LSTM, GRU, 1D-CNN and CNN-LSTM in the main 22/3/5 split.

Figure 6 presents the training histories of all six models in the main split. The curves belong to the 22/3/5 runs of Table 4, and the checkpoint with the lowest validation loss was used for testing. All models were trained for the full 100 epochs. The Transformer, 1D-CNN, GRU and CNN-LSTM reach a training accuracy of about 100% with a training loss close to zero, while the validation accuracy settles at roughly 95–98%. The Transformer’s validation accuracy rises to about 95% within approximately 15 epochs but then shows two short-lived drops with matching spikes in the loss (around epochs 22 and 37), after which it remains stable at about 97% until epoch 100. The GRU shows one short drop in validation accuracy with a spike in the validation loss around epoch 30 and recovers within a few epochs, and the LSTM shows similar drops around epochs 36 and 92. The 1D-CNN and CNN-LSTM have smoother loss curves with noisy validation accuracy. The MLP converges slowest: its training accuracy is still rising at epoch 100, and its loss does not reach zero, which is consistent with its lowest accuracy. (The training curves are recorded with dropout active, so they lie below the evaluation-mode training accuracies of Table 4).

**Figure 7:**
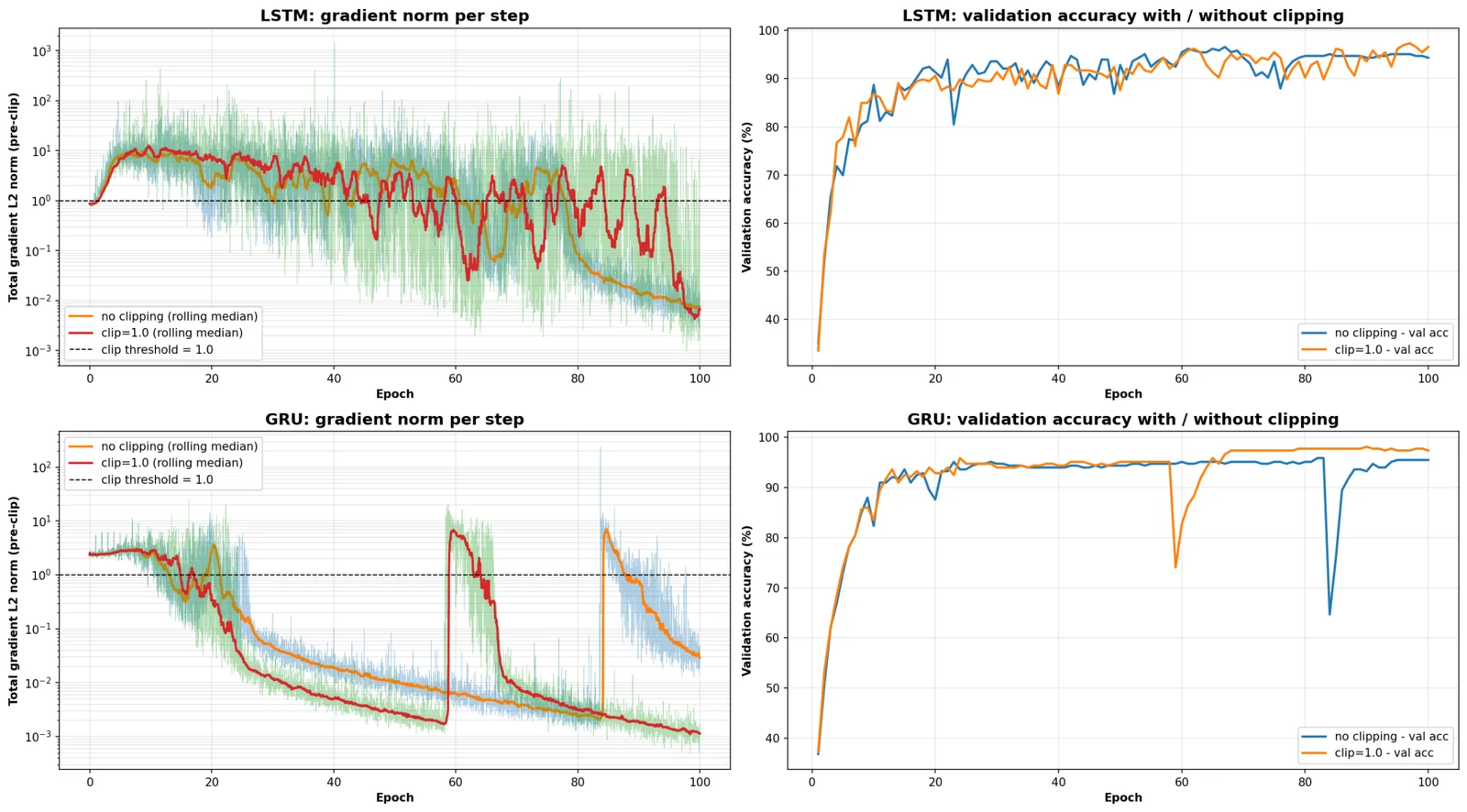
Gradient norm per training step (**left**; log scale, before clipping; thin lines indicate raw values, thick lines indicate rolling medians, and shaded areas indicate the interquartile range (IQR)) and validation accuracy (**right**) of the LSTM (**top**) and GRU (**bottom**) trained without clipping and with gradient-norm clipping at 1.0 (supplementary per-recording evaluation). The sharp drops in validation accuracy coincide with gradient-norm spikes.

**Training stability and gradient norms.** In the runs of Figure 6 none of the models collapses permanently; all show only transient spikes, and these occur in the Transformer as well as in the GRU and LSTM. To examine their cause, the LSTM and GRU were retrained in the supplementary per-recording evaluation with the total L2 norm of the gradients logged at every step (before clipping), once without clipping and once with gradient-norm clipping at 1.0 (Figure 7, Table 7). Three observations follow. (i) The large drops in validation accuracy coincide with gradient-norm spikes in the same epoch. Without clipping, the GRU’s validation accuracy fell from 95.9% to 64.7% at epoch 84, when the largest gradient norm in the epoch was 239, compared with maxima below 0.1 in the two preceding epochs; the LSTM’s validation accuracy fell from 94.0% to 80.5% at epoch 23 with a maximum norm of 164. (ii) The GRU collapses occurred after the training loss had approached zero (training accuracy 100%, median gradient norm 0.03 over the run). In this regime, the adaptive step size of Adam becomes large relative to the gradient, so that a single unusual mini-batch can produce a large update; this is consistent with an optimizer-related instability as well as with the exploding-gradient problem described for recurrent networks [19,45], and our measurements do not separate the two. (iii) Clipping did not prevent the collapses: in the clipped GRU run, the validation accuracy still fell from 95.1% to 74.1% at epoch 59, presumably because Adam rescales the clipped gradient. For the LSTM, whose gradient norm usually exceeded 1, 63% of the steps were clipped. Clipping reduced the number of validation drops of more than 5 points (LSTM: 5 to 2; GRU: 2 to 1) and gave slightly higher test accuracies (LSTM 93.23% to 95.74%; GRU 94.49% to 95.24%), but these are single runs whose differences are within the run-to-run variation in Section 4.2. We conclude that the transient collapses are associated with sudden gradient-norm spikes late in training, that clipping alone does not remove them, and that they did not affect the reported results, because the checkpoint with the lowest validation loss was used. A decaying learning rate or a larger ϵ in Adam are natural next remedies and remain to be tested.

### 4.4. Confusion Matrix Analysis

**Figure 8:**
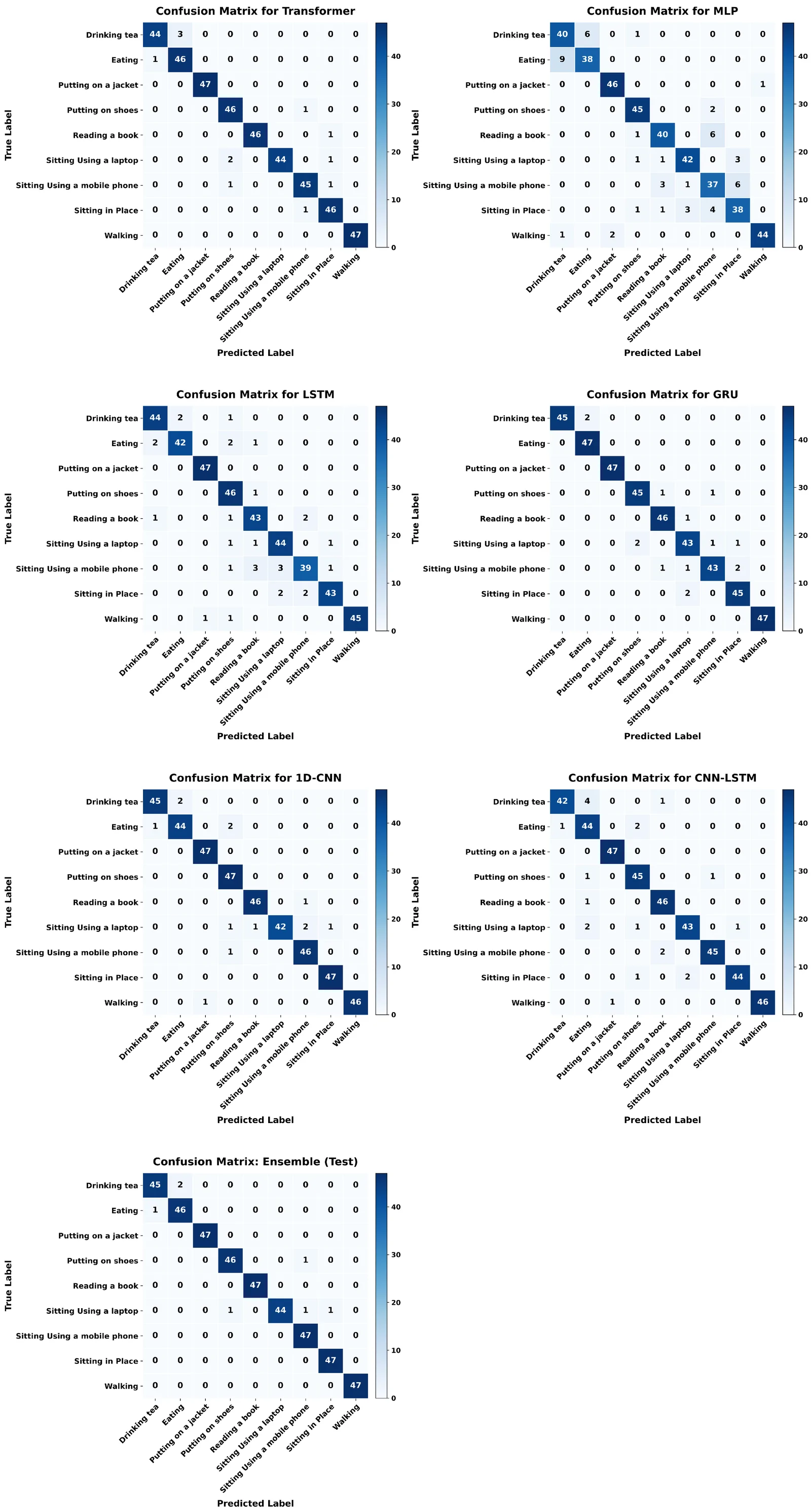
Confusion matrices on the held-out test participants (22/3/5 split, 47 samples per class) for the Transformer, MLP, LSTM, GRU, 1D-CNN, CNN-LSTM and the soft-voting ensemble. Rows represent true labels and columns represent predicted labels.

Figure 8 presents the confusion matrices of all six architectures and of the ensemble on the held-out test participants (47 samples per class, 423 in total), and Table 8 lists the corresponding per-class F1-scores. The matrices reproduce the accuracies of Table 4 (12, 21, 13, 30, 15 and 53 errors for the Transformer, CNN-LSTM, 1D-CNN, LSTM, GRU and MLP, and 7 for the ensemble). Activities involving large-scale body movements (walking and putting on a jacket) are recognized almost perfectly by all architectures (F1 of 95.7–100%, and 100% for the Transformer, the GRU and the ensemble), which is consistent with the strong radar signatures of gross motor activities. No single architecture is best for every seated activity: the Transformer has the highest F1 for reading a book (98.9%) and using a laptop (96.7%), the GRU for eating (97.9%) and drinking tea (97.8%), the 1D-CNN for sitting in place (98.9%) and putting on shoes (95.9%), and the CNN-LSTM for mobile phone use (96.8%). The ensemble has the highest or tied-highest F1 for seven of the nine classes (all except eating and drinking tea, where the GRU is higher by about one point).

**Figure 9:**
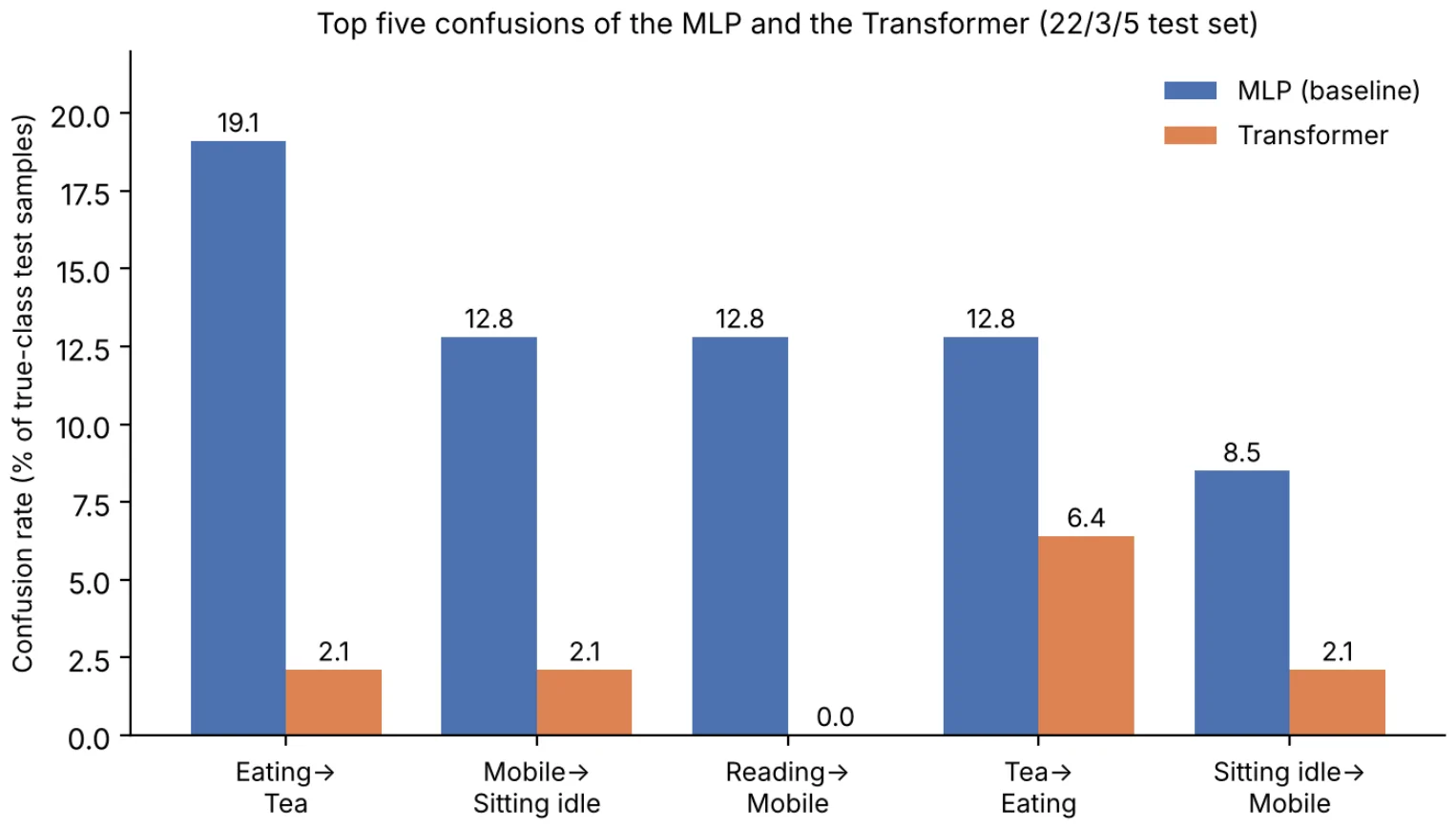
Top five inter-activity confusions of the MLP baseline (true class → predicted class) and the corresponding rates of the Transformer on the 22/3/5 test set.

The most frequently confused pair, summed over the six single models, is eating versus drinking tea (33 errors), followed by reading versus mobile phone use (18), mobile phone use versus sitting idle (17) and laptop use versus sitting idle (17). Eating and drinking tea share a repetitive hand-to-mouth movement at nearly the same range, and the other pairs share a stationary seated posture in which only subtle hand movements differ, while the variable timing of page turning produces inconsistent temporal signatures for reading (Figure 9). The MLP has the lowest F1-score for mobile phone use (77.1%) and for sitting in place (80.9%); it confuses eating with drinking tea in 19.1% of the eating samples, compared with 2.1% for the Transformer, and reading with mobile phone use in 12.8% of the reading samples, compared with 0%. The Transformer thus reduces confusion on the five most frequent MLP confusions by 50–100% (Figure 9).

**Figure 10:**
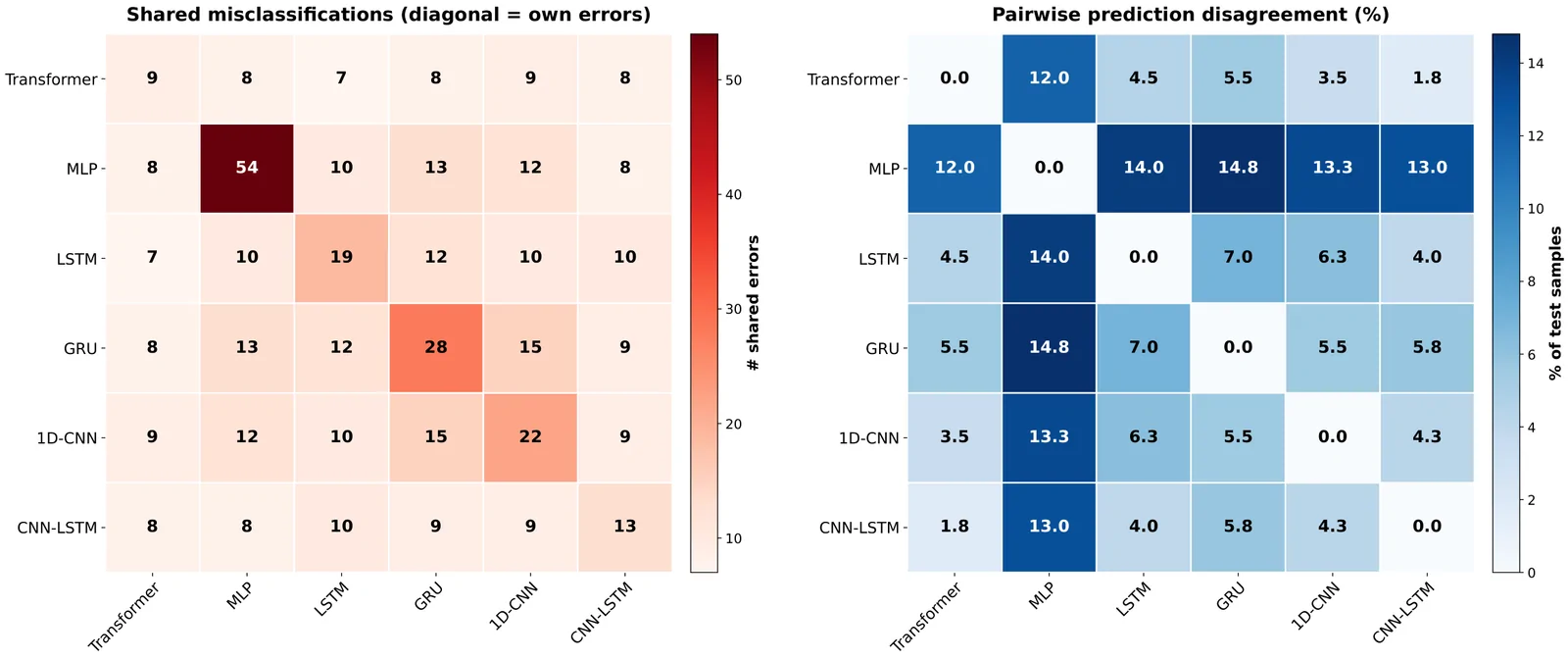
Sample-level error overlap of the six retrained models in the supplementary per-recording evaluation (399 test samples). Left: number of test samples misclassified by both models of a pair (diagonal: errors of the model itself). Right: percentage of test samples on which two models predict different classes.

**Error overlap between the models.** In the main split, the three ensemble members make 12 (Transformer), 13 (1D-CNN) and 15 (GRU) errors and the ensemble 7 (Table 4). All 7 ensemble errors occur between seated activities (eating versus drinking tea: 3; using a laptop: 3; putting on shoes versus mobile phone use: 1). At the level of confusion-matrix cells, the ensemble makes no error in any of the 11 cells in which only one member errs, and all its errors fall in cells in which at least two members err. To measure the overlap for individual samples, the per-sample predictions of the six retrained models of the supplementary per-recording evaluation were compared (399 test samples; Figure 10). In total, 82 samples (20.6%) are misclassified by at least one model: 58 by exactly one model, 11 by two, 1 by three, 4 by four, 2 by five and 6 by all six. Most errors are therefore model-specific, which is the precondition for ensembling. The errors of the strongest model, however, are not: all 9 errors of the Transformer are also made by the 1D-CNN, and 7–8 of them by each of the other models (Figure 10, left). The pairwise disagreement between the Transformer, CNN-LSTM, LSTM, GRU and 1D-CNN is 1.8–7.0% of the test samples, whereas the MLP disagrees with every other model on 12.0–14.8% (Figure 10, right). Consequently, the soft-voting ensemble of all six models (96.74%) did not improve on the Transformer (97.74%). It corrected none of the Transformer’s errors and introduced 4 new ones (exact McNemar test, p=0.125), although it was significantly better than the GRU (p<0.001), the 1D-CNN (p=0.012) and the MLP (p<0.001) and on par with the CNN-LSTM (p=1.0). An oracle that is correct whenever at least one model is correct would reach 98.50%, an upper bound for any combination of these six models. The six samples misclassified by all models are confusions between sitting idle and using a mobile phone (five samples) or reading (one sample), and four of them come from two participants (two recordings each); these residual errors may therefore reflect recording-specific behavior, such as holding a phone without visible interaction, rather than a weakness of a particular architecture. Together with the main-split result, this indicates that an ensemble helps only when the errors of its strongest member are not shared by the other members. Selecting the members on validation data, as done for the three-model ensemble, is therefore preferable to combining all models, and the 98.35% of the three-model ensemble should be regarded as a favorable case rather than an expected gain.

### 4.5. Activity Groups

Based on these results, the activities fall into two groups (Table 9). Walking and putting on a jacket involve substantial body displacement and produce strong, distinctive radar signatures, with an F1-score of at least 95.7% for every architecture (average 98.8%); even the MLP reaches 95.7–96.8% on them. The seven seated activities have a lower average F1-score of 93.1% and a much wider range across architectures (77.1–98.9%), and they are where the architectures differ. Within the seated group, we found no stable separation into easier and harder subgroups: the average F1 over the six architectures is lowest for mobile phone use (90.9%), eating (92.2%) and using a laptop (93.0%), and highest for putting on shoes (94.5%) and reading a book (94.3%), with sitting in place (93.2%) and drinking tea (93.3%) in between. This suggests that a simple architecture can detect gross activities, whereas attention-based, convolutional or gated recurrent sequence models, and preferably their ensemble, are needed for fine-grained seated activity analysis.

### 4.6. Computational Efficiency

Table 10 summarizes the computational cost of each architecture, measured in the supplementary evaluation in the Kaggle environment (Section 3.4). Latencies are medians of single-sample (batch size 1) forward passes after warm-up and exclude preprocessing; training times are CPU times per epoch, measured over three epochs. The 1D-CNN is the cheapest model on every measure: 0.88 M parameters (3.4 MB), 0.16 GFLOPs per sample, 1.9 ms per sample on the CPU and 0.66 ms on the GPU, and 7.8 s per training epoch on the CPU. It is about five times faster than the Transformer on the CPU (10.3 ms) while reaching a test accuracy within one sample of it in the main split, which makes it *the most promising candidate* for real-time or resource-constrained deployment. On the CPU the recurrent models are slow (13.5 ms for the LSTM and 25.9 ms for the GRU) because their 120 time steps are processed sequentially, and the MLP is the slowest model (29.5 ms) because of its 177 M-parameter input layer, although it needs few operations. The three-model ensemble requires running the Transformer, GRU and 1D-CNN (10.3 M parameters; about 38 ms per sample on the CPU and 7.3 ms on the GPU if run sequentially). A separate single-thread measurement of the 1D-CNN gave a median latency of 2.8 ms (3.0 ms including standardization), i.e., about 360 samples per second on one CPU core.

**Scope of the deployment claim.** These timings were measured on a server CPU and GPU, not on embedded hardware (the Raspberry Pi of the setup was used only for data acquisition), so no on-device latency or energy figures are available, and we describe the 1D-CNN as a promising candidate rather than a validated real-time solution. The figures nevertheless bound the cost. Each sample spans a 15 s observation window, so even the slowest model on the CPU (MLP, 29.5 ms) uses about 0.2% of the window, and the 1D-CNN would remain far below the window length on a processor many times slower than the server core. The preprocessing is not negligible in comparison: a one-dimensional wavelet-denoising stage (Section 4.8) took 6.6–17.1 ms per sample, more than the 1D-CNN itself, and computing a range–Doppler map took 9.6 ms. In 32-bit floating point, the weights of the 1D-CNN (3.4 MB) and the Transformer (21.6 MB) fit easily within the memory of single-board computers, whereas those of the MLP (677 MB) do not; 16-bit or 8-bit quantization could reduce storage further but was not evaluated.

### 4.7. Statistical Significance and Effect Size

Table 11 compares the Transformer with every other architecture using paired *t*-tests over the five cross-validation folds, Cohen’s *d* computed with the pooled fold standard deviation, and the difference in macro-averaged area under the ROC curve (AUC). The Transformer’s advantage is significant against all architectures (p<0.001), and all effect sizes are very large (d>2). The macro-averaged AUC of the Transformer is 0.9961, compared with 0.9950 for the CNN-LSTM, 0.9942 for the 1D-CNN, 0.9927 for the LSTM, 0.9916 for the GRU, and 0.9897 for the MLP.

**Reliability of the significance levels.** The five folds are drawn from the same 22 training participants and their training sets overlap, so the fold accuracies are positively correlated and the naive paired *t*-test underestimates the variance of the difference; with only four degrees of freedom the test is also sensitive to a single unusual fold. We therefore re-analyzed the fold accuracies of Table 3 (no new experiments) with three complementary procedures (Table 12). (i) The corrected resampled *t*-test of Nadeau and Bengio [44] inflates the variance of the mean difference by the factor $(1/k+n_{\mathrm{test}}/n_{\mathrm{train}})=1/5+1/4=0.45$ instead of 1/5 (statistical comparison of learning algorithms is also discussed by Dietterich [46]); all comparisons remain significant (the smallest effect, versus the CNN-LSTM, gives p≈0.001). (ii) The 95% confidence intervals of the mean differences, computed with the same correction, exclude zero for every comparison. (iii) The Transformer is better than each competitor in all five folds; the exact two-sided Wilcoxon signed-rank (equivalently sign) test with n=5 has a minimum attainable *p* of 0.0625, so the rank-based evidence cannot reach p<0.05 with five folds regardless of the effect size, and we report it only as consistency (5/5 folds). Overall, the cross-validation evidence supports the statement that the Transformer is the most accurate of the six *in-distribution* models of this study, with the largest uncertainty, as expected, for the closest competitor (the CNN-LSTM). None of these procedures can replace independent data: they quantify fold-to-fold variability on the same participants and say nothing about new participants. For this reason, the held-out results of Table 4, where the Transformer and 1D-CNN are statistically indistinguishable, remain the primary evidence about generalization.

### 4.8. Ablation Studies

Ablation studies were conducted on the Transformer to validate the architectural, preprocessing, and training choices; all values are five-fold cross-validation accuracies. Table 13 examines the architectural hyperparameters and the contribution of individual components. Increasing the number of attention heads from 2 to 8 improves accuracy by 2.27 percentage points, while 16 heads slightly degrade performance. Accuracy improves markedly from two to four encoder layers (+3.02 points), with only marginal gains beyond four layers, and an embedding dimension of 256 yields a 2.65-point gain over 128, whereas 512 dimensions add only 0.18 points at about 2.4 times the parameter count. The four-layer, eight-head, 256-dimensional configuration therefore offers a good accuracy–efficiency trade-off. Among the components, background subtraction contributes the most (6.04 points), followed by residual connections (4.91 points), wavelet denoising (4.53 points), and the feed-forward network (4.16 points); the 3.59-point contribution of positional encoding indicates the importance of temporal ordering.

Table 14 reports the preprocessing and training ablations. The two-dimensional sym5 wavelet denoising of the final pipeline improves accuracy by 4.53 points over the background-subtracted signal without denoising and outperforms Gaussian smoothing and median filtering, possibly because the wavelet thresholding suppresses broadband noise while preserving the short, localized echoes of hand and arm motions. Comparisons of wavelet families and decomposition levels made during development used an earlier one-dimensional configuration of the pipeline and do not correspond to the final setting, so they are not reported; the sym5 wavelet, level 2 and the multiplier of 1.5 should therefore be regarded as an empirically chosen configuration rather than an optimized one. A learning rate of $10^{-4}$ balances convergence speed and final accuracy, and a batch size of 32 achieves the highest accuracy, with larger batches degrading slightly, possibly because reduced gradient noise weakens generalization. These mechanistic explanations are plausible interpretations and were not tested separately. A dropout sweep made during development was run on an earlier version of the Transformer with a dropout layer in the classification head and does not correspond to the final model, which uses the default dropout of 0.1 inside the encoder layers and no separate classifier dropout; it is therefore not reported.

**Wavelet settings re-examined.** Because the wavelet-family and level ablations of the development pipeline do not correspond to the final setting, the wavelet stage was re-examined in the supplementary per-recording evaluation with the 1D-CNN, the cheapest model, on the held-out test set of that run (Table 15). Starting from the final pipeline (background subtraction, two-dimensional sym5 denoising at level 2 and standardization), an *additional* one-dimensional wavelet denoising along fast time (range) at level 3 was applied with five different wavelets, and each variant was trained once. The final pipeline alone reached 95.49%. None of the additional denoising stages improved on it by more than one test sample (Haar, 95.74%); the biorthogonal 3.5 wavelet was 1.25 points lower, and the db4, sym8 and coif5 wavelets reduced accuracy by 3.0–6.5 points, possibly because a second, stronger denoising with longer and smoother wavelets removes part of the fine range structure that distinguishes the seated activities. A second training run of the 1D-CNN on the same final pipeline gave 94.49% (Table 6), i.e., the run-to-run variation is about one point. These results indicate that the two-dimensional sym5 stage already removes most of the noise that wavelet thresholding can remove, and that further denoising is unnecessary and can be harmful. They do not re-measure the gain of the sym5 stage over no denoising, which was quantified only in the Transformer cross-validation ablation (4.53 points, Table 14); background subtraction remained the most important component in that ablation (Table 13).

### 4.9. Comparison with Previous Work

Table 16 compares the proposed models with recent radar-based activity recognition studies. This comparison is indicative only and must not be read as a ranking. The studies differ in the number and type of activities, in radar hardware and number of radars, in input representation, in the number and diversity of participants, and in evaluation protocol (e.g., random sample splits versus participant-independent splits). Accuracy is strongly affected by these factors: an accuracy of 98% on gross motions [11] and 96.93% on seven near-identical seated activities describe problems of different difficulty, and the lower figures of some studies partly reflect harder settings (e.g., distributed radars with arbitrary directions [14]). The numerical ranges are provided for context only; without a common dataset and evaluation protocol, neither superiority nor equivalence between systems can be inferred. None of the cited systems was re-implemented on our data, so a like-for-like comparison remains to be done.

#### Comparison with Domain-Specific UWB Gesture/Activity Architectures

**Figure 11:**
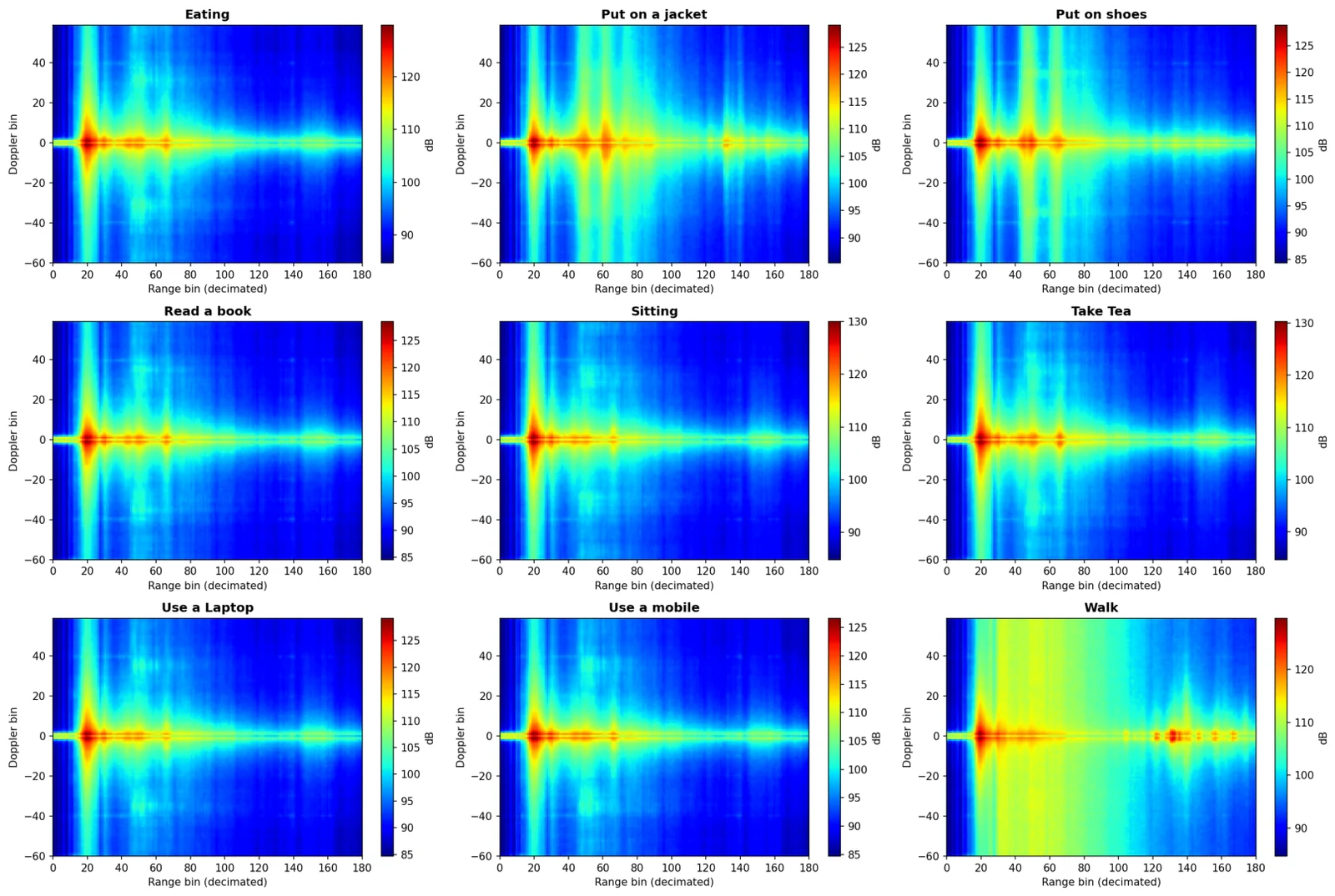
Mean range–Doppler map (dB) of each of the nine activities, i.e., the input of the range–Doppler baseline averaged over all samples of the class (Doppler axis in FFT bins). Apart from walking and putting on a jacket, the maps of the seated activities are very similar.

A relevant domain-specific model is the range–Doppler dual-channel fusion vision transformer for UWB-radar traffic-gesture recognition [33]. A full re-implementation of that network was not attempted, but a range–Doppler baseline was trained for this study to test whether a Doppler representation is informative for our activities. For each sample, an analytic signal was formed along fast time (Hilbert transform, so that the sign of the Doppler shift is kept), static clutter was removed by subtracting the slow-time mean of every range bin, and a Hann-windowed Fourier transform over the 120 slow-time frames gave 120 Doppler bins; the power was averaged over groups of 8 adjacent range bins (1440 to 180) and converted to dB. The resulting 120×180 maps were classified by a compact two-dimensional CNN (four convolutional blocks with 16–128 filters, global average pooling and dropout 0.5; RD-2DCNN, 98,793 parameters) trained with the same optimizer, learning rate, number of epochs and model selection as the other models (Figure 11). The baseline reached only 50.63% test accuracy (macro-F1 48.43%) on the 399 test samples of the re-run (Table 6). It recognized the gross-motion activities well (F1 97.8% for walking and 96.6% for putting on a jacket) and putting on shoes reasonably (82.5%), but the six other seated activities only with F1-scores of 4–37%. The maps of these activities are almost identical (Figure 11), presumably because a seated person produces mainly near-zero Doppler energy at the same range. Its training accuracy was still rising at epoch 100 (about 50%), so a larger or better-tuned Doppler model would likely do better, and the result does not show that Doppler-based architectures in general are unsuitable. It does indicate, however, that a range–Doppler map, which summarizes the whole slow-time signal by its spectrum, discards much of the temporal structure that distinguishes the seated activities and that the sequence models exploit directly from the CIR matrix. The specific traffic-gesture network was not re-implemented because (i) it was designed for large, fast, deliberate hand gestures; (ii) its main ingredients (embedding, multi-head self-attention and convolutional front ends) are already represented by our Transformer and CNN-LSTM, and our ablation (Table 13) shows the contribution of the attention components in our setting; and (iii) our conclusions are restricted to the evaluated model families, and we do not claim that the evaluated models are state-of-the-art for UWB gesture recognition. Architectures that fuse Doppler and time-domain representations remain an option for future work, and releasing the dataset (Data Availability) will allow others to test them.

## 5. Discussion

**Main findings.** The results indicate that attention-based, convolutional and gated recurrent architectures are well suited to fine-grained activity recognition from UWB radar, whereas the fully connected MLP is clearly inferior. The Transformer obtained the highest accuracy in each cross-validation fold (97.74% mean) and was also the best single model on the five held-out participants (97.16%), which may be due to the self-attention module’s ability to relate all 120 time steps to each other directly, an interpretation we did not test directly. Its margin over the 1D-CNN (96.93%) and the GRU (96.45%) on the test participants is only one and three samples. This is plausible for radar data: the discriminative information of a seated activity is concentrated in a few range bins around the subject and in short-lived hand motions, which small convolutional kernels with shared weights can capture efficiently, and the 1D-CNN does so with about one sixth of the parameters and about one fifth of the CPU inference time of the Transformer. The additional participant splits and the supplementary evaluation (Section 4.2) show that the 1D-CNN is the most stable single model across participant splits, whereas the Transformer loses accuracy as training participants decrease; no single architecture is uniformly best, and the changes between participant groups (up to 6 points for one model) are larger than most differences between models in a single split. Evaluation on unseen participants is therefore essential, together with an honest statement of its uncertainty.

**Ensemble.** The soft-voting ensemble of the Transformer, GRU and 1D-CNN reached the highest test accuracy in the main split (98.35%, 7 errors against 12, 15 and 13 for its members), was also best on the 20/3/7 split and was within 0.12 points of the best single model on the 17/3/10 split. The sample-level analysis of the re-run (Section 4.4) qualifies this result: most errors are made by a single model, but the errors of the strongest model (the Transformer) were shared by the other models, and an ensemble of all six models was one point less accurate than the Transformer alone. Ensembling is therefore not a reliable gain on this dataset: it can help when the members are selected on validation data and their errors are complementary, and its gain is small compared with the uncertainty of the test sets. The three-model ensemble also costs about five times the GPU inference time of the Transformer (7.3 ms versus 1.4 ms per sample) and about 3.7 times its CPU time (38 ms versus 10.3 ms). Since a 15 s observation window dominates the latency, this cost is acceptable on the hardware used here, whereas a single 1D-CNN remains the lighter option on constrained devices.

**Recurrent models.** The GRU reached 96.45% and the LSTM 92.91% on the test participants of the main split, and 92.98% and 95.24% in the supplementary evaluation, which shows how strongly their results vary between training runs. The gradient-norm logs (Section 4.3) show that their transient collapses coincide with sudden gradient-norm spikes after the training loss has approached zero, and that gradient clipping alone did not prevent them; similar transient drops also occurred in the Transformer. Because the checkpoint with the lowest validation loss was used, the collapses did not affect the reported accuracies. Even so, accurate recognition was achieved with a single radar module, which requires fewer sensors than multi-radar systems [10,14] and may simplify installation in homes.

### 5.1. Practical Deployment: Controlled Setting Versus Real Homes

All data were collected in a controlled indoor setting: a single seated or walking person, one room, one chair, a fixed radar height (72 cm), a fixed radar–subject distance (2 m), a fixed orientation toward the radar, and a static background. Real domestic environments differ in several respects that are relevant to the proposed pipeline. (i) *Distance and orientation.* A different distance shifts the activity to other range bins and changes the received power, and a different body orientation changes the radar cross-section and the visible limb motion; models trained at 2 m facing the radar may therefore degrade. The convolutional models share filters across range and may tolerate range shifts better than the fully connected MLP, but this was not tested. (ii) *Clutter and background.* Moving furniture, pets, fans, curtains and other people create time-varying reflections that violate the static-background assumption of the exponential-moving-average subtraction, which was the single most important pipeline component in our ablation (6.04 points, Table 13); the pipeline is therefore likely to be sensitive to such changes. (iii) *Multiple people and activity transitions.* Our samples contain one person performing one activity for 15 s, whereas real homes involve concurrent people, transitions and unlabeled activities. (iv) *Population.* All participants were healthy adults aged 18–45 years; older adults, who are the target group of ambient assisted living, move more slowly and with different amplitudes. For these reasons, the results should be read as a proof of feasibility under controlled conditions, not as a prediction of in-home accuracy. We did not run additional experiments with other distances, orientations or backgrounds, because they require new data collection beyond the scope of this study; these experiments are the most important next step.

### 5.2. Limitations

Several limitations should be acknowledged. All experiments were conducted on a single dataset collected in a controlled indoor environment at a fixed radar–subject distance, and the test set comprises a small number of participants (five in the main split; two further participant splits with 7 and 10 test participants (Table 5) show that accuracy can change by several points between participant groups, but they use fewer training participants and have not been analyzed with confidence intervals); cross-dataset validation and larger participant cohorts would strengthen the generalizability of the conclusions. The activity protocol does not include safety-critical events such as falls [47] or multi-person interactions, and real deployments may involve varying distances, orientations, and environmental clutter (Section 5.1). The statistical tests rely on only five cross-validation folds drawn from the same participants (mitigated, but not removed, by the corrections in Section 4.7). The supplementary analyses (error overlap, gradient clipping, wavelet comparison and range–Doppler baseline) were each run once, on a separate per-recording split with 399 test samples rather than on the main split; remedies for the training instabilities other than clipping (a learning-rate schedule, a larger Adam ϵ) were not tested; the computational figures were measured on a server CPU and GPU rather than on embedded hardware; and only a simple range–Doppler baseline, not a specialized fusion architecture, was benchmarked. Finally, although the Transformer and 1D-CNN are compact (5.7 M and 0.88 M parameters), deployment on low-power edge devices may require model compression or knowledge distillation, and the learned representations remain difficult to interpret, motivating explainable artificial intelligence techniques tailored to radar signals.

## 6. Conclusions

This paper compared six deep learning architectures and a soft-voting ensemble for single-radar, UWB-based recognition of nine daily activities, seven of which are fine-grained activities performed in the same seated position. With a preprocessing pipeline of background subtraction and two-dimensional Symlet-5 wavelet denoising, the Transformer achieved the highest five-fold cross-validation accuracy (97.74%). On five held-out participants the Transformer (97.16%), the 1D-CNN (96.93%) and the GRU (96.45%) performed similarly and ahead of the CNN-LSTM (95.04%), the LSTM (92.91%) and the MLP (87.47%), and the ensemble of the Transformer, GRU and 1D-CNN reached 98.35%. Two further participant splits and a separate supplementary evaluation with retrained models confirmed the weakness of the MLP and the strong but not uniformly best performance of the Transformer and the 1D-CNN, and showed variations of several points between runs, so the results should be regarded as indicative. In the supplementary per-recording evaluation, the errors of the Transformer were shared by the other models, so that an ensemble of all six models did not outperform it, and a simple range–Doppler baseline reached only 50.63%, which underlines the value of the temporal structure of the CIR matrix for seated activities. Gross-motion activities were recognized almost perfectly, while eating versus drinking tea and mobile phone use versus reading or sitting idle remained the most confusable pairs. The transient training collapses of the recurrent models coincided with gradient-norm spikes and were not removed by gradient clipping. These findings suggest that attention-based, convolutional and gated recurrent models are suitable for this type of data, with the 1D-CNN a promising low-cost candidate (1.9 ms per sample on a server CPU) for resource-constrained use, subject to the limitations above, in particular the small test cohort, the controlled setting and the absence of on-device evaluation. Future work will evaluate larger and more diverse participant groups, other distances, orientations and environments, leave-one-subject-out evaluation, learning-rate schedules for the recurrent models, architectures that fuse Doppler and time-domain representations, ensembles of complementary models, and on-device inference.

## Figures and Tables

**Table 1:** Focused comparison of related UWB radar studies by activity category, radar configuration and input representation. Information as reported by the original authors.

Study	Activity Category (no.)	Radar Configuration	Input Representation	Method (Reported Accuracy)
Bouchard [3]	Daily activities (15)	Three XeThru X4M200 UWB radars	Hand-crafted statistical and wavelet-energy features per radar	Random forest (80.0%)
Maitre [10]	Daily activities (15)	Three XeThru X4M200 UWB radars	One normalized 1-D time series per radar (scattering matrix summed over range; 15 s windows)	CNN–LSTM (94%; voting 95%)
Du [15]	Various motions	UWB radar	Micro-Doppler spectrograms	Segmented CNN-GRU (study-specific results)
Guendel [14]	Activities, arbitrary directions (9)	Five distributed P410 UWB radars	Hand-crafted features of range–Doppler maps	Softmax + feature fusion (study-specific results)
Qi [11]	Gross human motions (12)	Single XeThru X4M300 IR-UWB radar	Radial features (KNN), then power-spectrum and Doppler-shift spectrograms	KNN + GoogLeNet (98.0%)
Lafontaine [31]	Daily activities (14)	UWB radar	CIR data, denoised	CNN autoencoder + CNN (69.9%)
Wang [12]	Hidden (through-wall) activities (11)	UWB radar	Complex-valued range profiles	CV-MCNet (study-specific results)
Wang [8]	Through-wall activities (11)	UWB radar	Multidomain fusion	GMFN, CNN+GCN (study-specific results)
Zhang [5]	Vital signs during physical activity (5 activities)	Two Novelda X4M03 IR-UWB radars (chest and abdomen)	EWT-decomposed chest and abdomen signals	Cross-correlation (86.9% heart-rate estimation accuracy)
Xiong [33]	Traffic gestures	UWB radar	Range–Doppler frequency and intensity maps (dual channel)	DCF-ViT (no overall accuracy in abstract)
This work	**9 activities, 7 seated in one posture**	**Single monostatic P410, 2 m**	**Preprocessed raw CIR matrix (120×1440)**	**Six models + ensemble (97.74% CV; up to 98.35% test)**

**Table 2:** Deep learning model architectures summary.

Model	Key Layers	Architecture Description
Transformer	Linear embedding, learnable positional encoding, multi-head self-attention, feed-forward network, global average pooling, linear classifier	Encoder-only; $d_{\mathrm{model}}=256$, 8 heads, 4 layers, feed-forward width 2048; dropout 0.1 (encoder layers)
CNN-LSTM	Conv1D, BatchNorm, MaxPool1D, bidirectional LSTM, dense	Conv1D (128 filters, kernel 3) + pooling, then 2-layer Bi-LSTM (256 units); dropout 0.2
1D-CNN	Conv1D, ReLU, BatchNorm, MaxPool1D, global average pooling, dense	3 blocks (128, 256, 256 filters, kernel 3), dense 128, dropout 0.5
LSTM	Bidirectional LSTM, dense	2 bidirectional layers (256 units); final hidden states of both directions; dropout 0.2
GRU	Bidirectional GRU, dense	2 bidirectional layers (256 units); final hidden states of both directions; dropout 0.2
MLP	Flatten, dense, ReLU, dropout	Dense 1024 and 512 units, dropout 0.5
Ensemble	Trained Transformer, GRU and 1D-CNN (main split), softmax probability averaging	Soft voting over class probabilities of the three best validation models with equal weights; no additional trainable parameters

**Table 3:** Five-fold cross-validation accuracy (%) on the training participants.

Model	Fold 1	Fold 2	Fold 3	Fold 4	Fold 5	Mean ± Std	95% CI
Transformer	97.52	98.11	97.36	97.92	97.79	97.74 ± 0.30	[97.37, 98.11]
CNN-LSTM	96.23	97.17	96.42	96.79	96.42	96.61 ± 0.38	[96.14, 97.07]
1D-CNN	95.47	96.23	95.66	96.04	95.85	95.85 ± 0.30	[95.48, 96.22]
LSTM	93.96	94.72	94.15	94.53	94.34	94.34 ± 0.30	[93.97, 94.71]
GRU	92.83	93.58	93.21	93.40	93.02	93.21 ± 0.30	[92.84, 93.58]
MLP	90.94	91.70	91.13	91.51	91.32	91.32 ± 0.30	[90.95, 91.69]

**Table 4:** Performance of individual models and the soft-voting ensemble on the training, validation and held-out test participants (22/3/5 split; 423 test samples). Precision, recall and F1 are macro-averaged. Errors = misclassified test samples; CI = 95% Wilson score interval of the test accuracy. The ensemble combines the Transformer, GRU and 1D-CNN.

Model	Train acc.	Val. acc.	Test acc.	Test prec.	Test Recall	Test F1	Errors	95% CI (%)
Transformer	100.00	97.53	97.16	97.24	97.16	97.17	12	[95.11, 98.37]
CNN-LSTM	100.00	95.88	95.04	95.23	95.04	95.06	21	[92.53, 96.73]
1D-CNN	100.00	96.71	96.93	97.02	96.93	96.91	13	[94.81, 98.20]
LSTM	99.54	94.65	92.91	93.06	92.91	92.90	30	[90.06, 94.99]
GRU	100.00	96.71	96.45	96.48	96.45	96.45	15	[94.23, 97.84]
MLP	97.13	89.30	87.47	87.60	87.47	87.49	53	[83.97, 90.29]
Ensemble (T + GRU + 1D-CNN)	100.00	97.94	**98.35**	98.37	98.35	98.34	7	[96.62, 99.20]

**Table 5:** Test accuracy (%) of all models for three participant-wise splits (training/validation/test participants; numbers of test samples in parentheses). Mean ± SD and range are over the three splits (SD with n−1). In each split, the ensemble combines the three models with the highest validation accuracy (Transformer + GRU + 1D-CNN for 22/3/5; CNN-LSTM + Transformer + 1D-CNN for 20/3/7 and 17/3/10).

Model	22/3/5 (423)	20/3/7 (603)	17/3/10 (873)	Mean ± SD	Range
Transformer	97.16	94.69	90.95	94.27 ± 3.13	6.21
CNN-LSTM	95.04	94.03	91.98	93.68 ± 1.56	3.06
1D-CNN	96.93	95.69	94.39	95.67 ± 1.27	2.54
LSTM	92.91	95.02	89.23	92.39 ± 2.93	5.79
GRU	96.45	94.03	93.24	94.57 ± 1.67	3.21
MLP	87.47	87.56	86.60	87.21 ± 0.53	0.96
Ensemble (3 models)	98.35	96.68	94.27	96.43 ± 2.05	4.08

**Table 6:** Test accuracy of the retrained models in the supplementary per-recording evaluation (per-recording split, 399 test samples, 44–45 per class) compared with the participant-independent main split (Table 4, 423 test samples). The re-run ensemble combines all six models; the oracle counts a sample as correct if at least one of the six models is correct.

Model	Main Split: Test acc. (%)	Re-Run: Test acc. (%)	Re-Run: Errors (of 399)
Transformer	97.16	**97.74**	9
CNN-LSTM	95.04	96.74	13
LSTM	92.91	95.24	19
1D-CNN	96.93	94.49	22
GRU	96.45	92.98	28
MLP	87.47	86.47	54
Range–Doppler 2D-CNN (Section Comparison with Domain-Specific UWB Gesture/Activity Architectures)	–	50.63	197
Ensemble, 3 models (T + GRU + 1D-CNN), soft voting	98.35	–	–
Ensemble, all 6 models, soft voting	–	96.74	13
Ensemble, all 6 models, hard voting	–	96.74	13
Oracle (any of the 6 models correct)	–	98.50	6

**Table 7:** LSTM and GRU trained with and without gradient-norm clipping (supplementary per-recording evaluation, 399 test samples, one run each). Gradient norms are total L2 norms per step before clipping; drops = epochs in which the validation accuracy fell by more than 5 points.

Model	Clipping	Test acc. (%)	Macro-F1 (%)	Best Epoch	Median Norm	Max Norm	Steps Clipped (%)	Drops
LSTM	none	93.23	93.22	61	1.880	163.6	–	5
LSTM	max-norm 1.0	95.74	95.70	100	2.531	1410.6	62.6	2
GRU	none	94.49	94.46	35	0.028	239.0	–	2
GRU	max-norm 1.0	95.24	95.19	69	0.007	26.3	21.6	1

**Table 8:** Per-class F1-scores (%) of all architectures on the held-out test participants (computed from the confusion matrices of Figure 8).

Activity	Transformer	CNN-LSTM	1D-CNN	LSTM	GRU	MLP	Ensemble
Eating	95.8	88.9	94.6	92.3	97.9	83.5	96.8
Putting on a jacket	100.0	98.9	98.9	98.9	100.0	96.8	100.0
Putting on shoes	95.8	93.8	95.9	92.0	95.7	93.8	97.9
Reading a book	98.9	95.8	97.9	89.6	96.8	87.0	100.0
Sitting in place	95.8	95.7	98.9	93.5	94.7	80.9	98.9
Drinking tea	95.7	93.3	96.8	93.6	97.8	82.5	96.8
Sitting using a laptop	96.7	93.5	94.4	91.7	91.5	90.3	96.7
Sitting using a mobile phone	95.7	96.8	95.8	86.7	93.5	77.1	97.9
Walking	100.0	98.9	98.9	97.8	100.0	95.7	100.0
**Macro average**	97.2	95.1	96.9	92.9	96.4	87.5	98.3

**Table 9:** Activity groups; F1 range and average are taken over the six single architectures (Table 8).

Group	Activities	Radar Signal Characteristics	F1 Range (%)	Avg. F1 (%)
Gross motion	Walking, putting on a jacket	Large body displacement; strong, distinctive motion signatures	95.7–100	98.8
Seated (7)	Eating, drinking tea, putting on shoes, reading a book, sitting using a laptop, sitting using a mobile phone, sitting in place	Minimal torso movement; weak, localized hand and arm motions with irregular timing	77.1–98.9	93.1

**Table 10:** Computational cost of the models, measured in the supplementary evaluation (Intel Xeon 2.00 GHz CPU, PyTorch with two threads; NVIDIA Tesla T4 GPU; PyTorch 2.11). Weights in MB ($2^{20}$ bytes, 32-bit floating point); latency = median single-sample (batch size 1) forward pass, preprocessing excluded; training = CPU time per epoch (mean of three epochs). The range–Doppler map computation (9.6 ms per sample) is not included for the RD-2DCNN.

Model	Params (M)	Weights (MB)	GFLOPs	CPU Latency (ms)	GPU Latency (ms)	CPU Training (s/Epoch)
Transformer	5.66	21.6	1.406	10.26	1.38	95.9
CNN-LSTM	2.93	11.2	0.416	7.14	3.04	17.9
1D-CNN	0.88	3.4	0.156	**1.91**	**0.66**	**7.8**
LSTM	5.06	19.3	1.211	13.48	5.15	45.6
GRU	3.80	14.5	0.908	25.88	5.22	49.4
MLP	177.48	677.0	0.355	29.46	2.72	102.9
RD-2DCNN (baseline)	0.10	0.4	0.154	4.68	0.74	18.4

**Table 11:** Statistical comparison of the Transformer with the other architectures (five-fold cross-validation).

Comparison	Mean Diff. (pp)	*t*	*p*	Cohen’s *d*	ΔAUC
Transformer vs. CNN-LSTM	1.13	12.87	<0.001	3.33	+0.0011
Transformer vs. 1D-CNN	1.89	33.31	<0.001	6.28	+0.0019
Transformer vs. LSTM	3.40	59.92	<0.001	11.29	+0.0034
Transformer vs. GRU	4.53	42.48	<0.001	15.13	+0.0045
Transformer vs. MLP	6.42	113.14	<0.001	21.32	+0.0064

**Table 12:** Additional statistical analysis of the five-fold cross-validation accuracies (Table 3); Transformer minus competitor. Corrected: Nadeau–Bengio variance correction. Folds won: number of folds in which the Transformer is better.

Comparison	Mean Diff. (pp)	95% CI (Corrected)	Corrected *t* (4 df)	Corrected *p*	Folds Won
vs. CNN-LSTM	1.13	[0.77, 1.50]	8.58	0.001	5/5
vs. 1D-CNN	1.89	[1.65, 2.13]	22.2	<0.001	5/5
vs. LSTM	3.40	[3.16, 3.64]	39.9	<0.001	5/5
vs. GRU	4.53	[4.09, 4.98]	28.3	<0.001	5/5
vs. MLP	6.42	[6.18, 6.66]	75.4	<0.001	5/5

**Table 13:** Transformer architecture and component ablation (five-fold cross-validation accuracy). Parameter counts are computed for the PyTorch implementation (feed-forward width 2048); the number of heads does not change the parameter count.

Configuration	Accuracy (%)	Params (M)	Time (s/Epoch)
*Number of attention heads*			
2 heads	95.47 ± 0.82	5.66	42.1
4 heads	96.61 ± 0.68	5.66	43.5
8 heads (proposed)	97.74 ± 0.30	5.66	45.3
16 heads	97.36 ± 0.59	5.66	48.7
*Number of encoder layers*			
2 layers	94.72 ± 0.91	3.03	24.8
6 layers	97.92 ± 0.48	8.29	66.2
8 layers	97.55 ± 0.62	10.92	87.4
*Embedding dimension $d_{\mathrm{model}}$*			
128	95.09 ± 0.78	2.57	28.6
512	97.92 ± 0.55	13.41	89.7
*Component removed from the proposed model (*Δ *accuracy in parentheses)*			
Positional encoding	94.15 ± 0.92 (−3.59)	–	–
Multi-head → single-head attention	95.47 ± 0.82 (−2.27)	–	–
Layer normalization	95.09 ± 0.88 (−2.65)	–	–
Feed-forward network	93.58 ± 1.05 (−4.16)	–	–
Residual connections	92.83 ± 1.18 (−4.91)	–	–
Background subtraction	91.70 ± 1.24 (−6.04)	–	–

**Table 14:** Preprocessing and training configuration ablation for the Transformer (five-fold cross-validation accuracy, %). The proposed setting is 97.74 ± 0.30 (two-dimensional sym5 wavelet, level 2, threshold multiplier 1.5; encoder dropout 0.1).

Preprocessing	Accuracy	Training	Accuracy (Conv. Epoch)
*Denoising method*		*Learning rate*	
No denoising	93.21 ± 1.12	$10^{-3}$	96.23 ± 0.85 (35)
Wavelet (2-D sym5, level 2)	**97.74**	$10^{-4}$	**97.74** (20)
Gaussian smoothing	94.34 ± 0.89	$10^{-5}$	96.79 ± 0.72 (65)
Median filtering	93.96 ± 0.95		
		*Batch size*	
		16	97.55 ± 0.68 (28)
		32	**97.74** (20)
		64	97.36 ± 0.58 (18)
		128	96.79 ± 0.71 (15)

**Table 15:** Additional wavelet denoising for the 1D-CNN on the held-out test set of the supplementary per-recording evaluation (399 samples, one run each). All rows start from the final pipeline (background subtraction, two-dimensional sym5 denoising at level 2, standardization); the variants add a one-dimensional decomposition along fast time (range) at level 3 with soft universal thresholding.

Additional Wavelet Stage	Test acc. (%)	Macro-F1 (%)	Best Epoch	Extra Time (ms/Sample)
None (final pipeline only)	95.49	95.46	59	–
Haar	**95.74**	**95.73**	75	6.6
db4	90.23	90.16	79	8.5
sym8	92.48	92.47	36	11.2
coif5	88.97	88.94	52	17.1
bior3.5	94.24	94.23	53	9.8

**Table 16:** Comparison with previous radar-based activity recognition studies. Values are as reported by the original authors on different datasets and protocols and are not directly comparable. ^*a*^ Heart-rate estimation accuracy, not activity classification accuracy.

Study	Activities	Method	Accuracy	Limitation Addressed in This Work
Bouchard [3]	15 ADLs	Random forest	80.00%	Hand-crafted features; no temporal deep learning
Maitre [10]	15 ADLs	CNN–LSTM + voting	95.00% (voting); 94.00% (CNN–LSTM)	Requires three radars; no attention mechanism
Du [15]	Various	SCGRNN	Not reproduced here	Single architecture; gross motor focus
Guendel [14]	9	Softmax + fusion	Not reproduced here	Five distributed radars; arbitrary directions
Qi [11]	12	KNN + GoogLeNet	98.00%	Two-stage pipeline; gross motor focus
Lafontaine [31]	14	CNN-AE + CNN	69.90%	Denoising focus; low final accuracy
Wang [12]	11	CV-MCNet	Not reproduced here	Single architecture; no sedentary focus
Wang [8]	11	GMFN (CNN + GCN)	Not reproduced here	Through-wall scenario only
Zhang [5]	5	Cross-correlation	86.90% ^*a*^	Vital-sign focus; few activities
This work	9 (7 seated)	Transformer	97.74% (CV); 97.16% (test)	Single radar; six-model comparison; unseen-participant evaluation
This work	9 (7 seated)	1D-CNN	96.93% (test)	Fastest inference (1.9 ms/sample on CPU)
This work	9 (7 seated)	Ensemble (T + GRU + 1D-CNN)	98.35% (test)	Three-model soft voting; no extra parameters

## Data Availability

The dataset used in this study is currently being utilized in an ongoing research project and is therefore not publicly available at this time. Upon completion of that project, the dataset is intended to be publicly released. In the interim, the data may be made available upon reasonable request to the corresponding authors, subject to ethical approval and data protection regulations.
